# Early peripheral monocyte dynamics are associated with 28-day mortality in acute liver failure: an exploratory longitudinal cohort study

**DOI:** 10.3389/fmed.2026.1945730

**Published:** 2026-09-10

**Authors:** Bin Niu, Rong Wang, Xinran Li, Haiyan Tian, Yangdi Yu, Qiongjie Li, Liaoyun Zhang

**Affiliations:** 1 Department of Infectious Diseases, The First Hospital of Shanxi Medical University, Taiyuan, China; 2 The First Clinical Medical School, Shanxi Medical University, Taiyuan, China

**Keywords:** acute liver failure, immune dysregulation, longitudinal dynamics, monocytes, mortality

## Abstract

**Background:**

Acute liver failure (ALF) is a rapidly progressive and life-threatening syndrome with high short-term mortality. The prognostic value of early longitudinal changes in peripheral immune cell counts remains unclear. This study investigated whether early dynamic changes in peripheral monocytes (MONO) are associated with 28-day mortality in ALF.

**Methods:**

In this single-center retrospective longitudinal cohort study, 266 patients with International Classification of Diseases-coded ALF between October 2018 and January 2026 were screened. After chart review and application of predefined diagnostic criteria, 46 patients were included. Peripheral blood cell parameters within 7 days after admission were analyzed. MONO-derived indices included delta MONO and slope MONO. Linear mixed-effects models assessed MONO trajectories. Additional analyses included clustering, logistic regression, and model evaluation.

**Results:**

Among 46 patients, 23 (50.0%) died within 28 days, while the remaining 23 patients survived. Baseline MONO did not differ between groups. MONO trajectories showed a stable pattern in survivors and a progressive increase in non-survivors, with significant time × group interaction after adjustment for age, gender, and white blood cells. Higher delta MONO and slope MONO were observed in non-survivors. Trajectory-based clustering identified an increasing-MONO phenotype associated with higher mortality. In multivariable analyses, delta MONO showed the most consistent association with 28-day mortality, whereas baseline MONO showed model-dependent associations and slope MONO was less stable after covariate adjustment. Exploratory prognostic analyses suggested that MONO dynamics may provide complementary information beyond conventional variables. A shorter-window analysis further showed that MONO trajectory divergence was already detectable within the first 72 h after admission.

**Conclusion:**

Early increases in peripheral MONO are associated with 28-day mortality in ALF, with delta MONO showing the most consistent association across multivariable analyses. Longitudinal MONO monitoring may provide complementary information beyond single baseline measurements, although these findings require validation in larger cohorts.

## 1 Introduction

Acute liver failure (ALF) is a rare but life-threatening clinical syndrome characterized by rapid deterioration of liver function in individuals without pre-existing cirrhosis, typically accompanied by coagulopathy and hepatic encephalopathy within a short period. Despite substantial improvements in intensive care management, artificial liver support, and liver transplantation strategies, ALF remains associated with considerable short-term mortality. The rapid progression of ALF and the frequent development of systemic complications, including infection, cerebral edema, and multiple organ dysfunction, make early identification of patients at high risk of deterioration essential for timely intervention and individualized clinical management (1).

The epidemiological characteristics and clinical outcomes of ALF vary substantially across different regions and etiological backgrounds. Viral hepatitis, drug-induced liver injury, autoimmune hepatitis, and other less common causes contribute to heterogeneous disease presentations and outcomes (2). Currently, several prognostic scoring systems, including the King's College Hospital criteria (KCC) and Model for End-Stage Liver Disease (MELD)-based models, are widely used to assist clinical decision-making and evaluate the need for transplantation (3). However, these approaches mainly rely on conventional indicators reflecting hepatic functional impairment, such as coagulation abnormalities and biochemical changes, and are usually assessed at a single time point. Given the dynamic nature of ALF progression, static measurements may not fully capture ongoing biological changes associated with disease deterioration. Therefore, identifying novel dynamic biomarkers capable of reflecting real-time disease evolution remains an important unmet clinical need.

Beyond the extent of hepatocellular injury itself, accumulating evidence indicates that immune dysregulation plays an important role in the clinical progression of ALF. Extensive liver injury can initiate complex interactions between damaged hepatocytes and immune cells, leading to activation of innate immune pathways and systemic inflammatory responses (4). While appropriate immune activation contributes to tissue repair and recovery, excessive or persistent inflammatory responses may aggravate organ dysfunction, whereas impaired immune regulation may increase susceptibility to secondary complications such as infection and sepsis (5). Therefore, characterization of immune alterations during the course of ALF may provide additional insights into disease progression and help identify potential prognostic indicators.

Increasing evidence supports a central role of immune dysregulation in the pathogenesis and progression of severe liver injury. Both innate and adaptive immune responses contribute to hepatocellular damage, systemic inflammation, and extrahepatic complications, and immune-related biomarkers have been explored as potential indicators of disease severity and prognosis in ALF (6, 7). However, routine clinical assessment and most previous biomarker studies have primarily focused on static measurements obtained at admission, while the prognostic significance of longitudinal immune-cell dynamics remains insufficiently defined.

Monocytes (MONO) are key mediators of innate immune responses and systemic inflammation in critical illness. Following acute liver injury, circulating monocytes may undergo dynamic changes associated with inflammatory activation, immune regulation, and disease progression. However, the temporal patterns of peripheral MONO changes and their relationship with outcomes in ALF have not been systematically evaluated. Given that dynamic alterations may better reflect ongoing pathophysiological processes than single baseline measurements, this study aimed to investigate early longitudinal MONO trajectories and their association with 28-day mortality in patients with rigorously defined ALF.

## 2 Materials and methods

### 2.1 Study design and ethical approval

This single-center retrospective longitudinal cohort study was conducted at the First Hospital of Shanxi Medical University. Patients with an International Classification of Diseases (ICD)-coded diagnosis of ALF between October 2018 and January 2026 were identified through the hospital information system. The sample size was determined by the availability of eligible patients during the study period, and all patients identified during the study period were included. All ICD-coded patients were reviewed according to predefined diagnostic criteria. Final ALF diagnoses were independently confirmed by two experienced clinicians through comprehensive chart review.

This study was approved by the Ethics Committee of the First Hospital of Shanxi Medical University (NO. KYYJ-2026–119). The requirement for informed consent was waived due to the retrospective design and use of previously collected clinical data.

### 2.2 Study subjects

Patients were included if they met the diagnostic criteria for ALF, defined as acute deterioration of liver function in the absence of pre-existing chronic liver disease, accompanied by coagulopathy and hepatic encephalopathy within the clinical course (8). Diagnosis was confirmed through detailed chart review, including medical history, laboratory tests, imaging findings, and disease progression.

Patients were excluded if they had acute-on-chronic liver failure, chronic liver failure, subacute liver failure not meeting ALF criteria, hypoperfusion-related liver injury, liver dysfunction secondary to infection, malignancy, or other advanced systemic diseases. Additional exclusion criteria included isolated coagulation abnormalities without evidence of liver failure and insufficient clinical data for diagnosis or outcome assessment.

**Figure 1:**
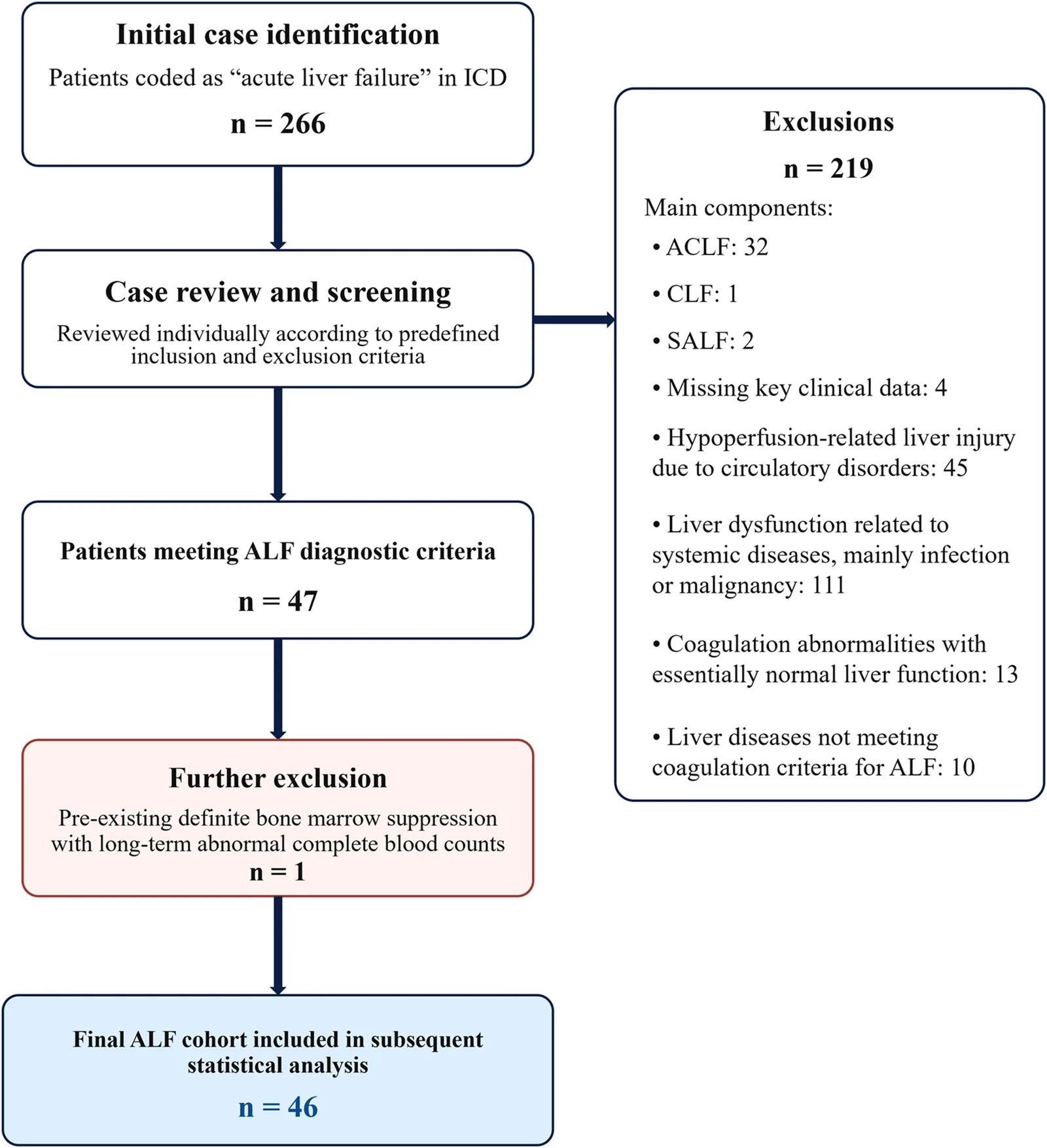
Flow diagram of patient screening, exclusion, and final cohort selection. A total of 266 patients with an International Classification of Diseases-coded diagnosis of acute liver failure (ALF) were initially identified. After detailed chart review according to predefined inclusion and exclusion criteria, 219 patients were excluded and 47 patients met the diagnostic criteria for ALF. One additional patient with pre-existing definite bone marrow suppression and long-term abnormal complete blood counts was excluded, leaving 46 patients in the final analytical cohort.

The detailed patient selection process is illustrated in Figure 1.

### 2.3 Data collection

Demographic characteristics, clinical manifestations, etiological information, laboratory findings, organ dysfunction parameters, therapeutic interventions, prognostic scores, and clinical outcomes were retrospectively extracted from the electronic medical record system. Data collection was performed according to a predefined standardized protocol to ensure consistency across patients.

Baseline demographic characteristics included age, gender, and underlying comorbidities. Clinical characteristics collected at admission included the department of initial hospitalization, intensive care unit (ICU) admission, presence of ascites, pleural effusion, gastrointestinal bleeding, infection-related complications, sepsis, renal dysfunction, respiratory failure, and other organ complications occurring during hospitalization. Information regarding organ support therapies, including mechanical ventilation, vasopressor support, continuous renal replacement therapy (CRRT), and artificial liver support, was also recorded.

Etiological information was collected based on clinical diagnosis and available laboratory investigations. Potential precipitating factors, including drug-induced liver injury, viral hepatitis, autoimmune-related causes, and other identifiable causes, were documented.

Peripheral blood cell parameters during the first 7 days after admission were specifically collected for longitudinal analyses, including white blood cells (WBC), neutrophils (NEUT), lymphocytes (LYMPH), MONO, hemoglobin (HGB), and platelets (PLT). When multiple measurements were available on the same day, the value closest to routine morning blood collection was selected to minimize potential influence from short-term fluctuations. All included patients had at least two available peripheral blood cell measurements during the first 7 days after admission, allowing assessment of individual-level temporal changes. The number of repeated measurements ranged from two to three per patient, reflecting routine clinical monitoring frequency during hospitalization.

To characterize dynamic MONO changes, two patient-level MONO-derived indicators were calculated. Delta MONO was defined as the absolute change between the last available MONO measurement within the first 7 days after admission and the baseline MONO value. Slope MONO was calculated using individual-level linear regression models, with MONO values as the dependent variable and days after admission as the independent variable, representing the average rate of MONO change over the observation period.

Additional inflammation-related composite indices were calculated, including neutrophil-to-lymphocyte ratio (NLR), monocyte-to-lymphocyte ratio (MLR), platelet-to-lymphocyte ratio (PLR), systemic immune-inflammation index (SII), and systemic inflammation response index (SIRI), according to previously established formulas (9–13).

Conventional prognostic scoring systems for ALF, including KCC, MELD, and MELD-Na scores, were calculated based on previously published algorithms (14–16).

### 2.4 Outcome definition

The primary outcome was 28-day mortality after admission. Patients were classified as survivors or non-survivors according to their survival status at day 28, as documented in the electronic medical records.

### 2.5 Longitudinal assessment of peripheral blood cell parameters

Longitudinal analyses were performed using repeated peripheral blood cell measurements obtained during the first 7 days after admission. This observation window was selected because early clinical deterioration and immune alterations are considered critical determinants of short-term outcomes in ALF.

Longitudinal trajectories of WBC, NEUT, LYMPH, MONO, HGB, and PLT were evaluated separately between survivors and non-survivors. Additional exploratory longitudinal analyses were performed for inflammatory indices, coagulation parameters, and liver function-related markers to determine whether similar temporal patterns existed beyond MONO.

### 2.6 Linear mixed-effects model

Linear mixed-effects models (LMM) were applied to formally evaluate differences in MONO trajectories between survivors and non-survivors while accounting for repeated measurements within the same individual.

The fixed effects included time, survival group, and the interaction term between time and survival group. The time-by-group interaction was considered the primary parameter of interest, reflecting whether the temporal pattern of MONO changes differed between survivors and non-survivors. Individual patient identification numbers were included as random effects to account for within-subject correlations caused by repeated measurements.

To evaluate the robustness of the observed associations, sequential adjustment models were constructed. The primary model was adjusted for age, gender, and baseline WBC, considering their potential influence on circulating immune-cell dynamics. The estimated regression coefficients, 95% confidence intervals, and corresponding *P* values were reported.

### 2.7 Association analysis of MONO-derived indicators and trajectory-based clustering

To evaluate the association between MONO dynamics and 28-day mortality, multivariable logistic regression analyses were performed using MONO-derived indicators as independent variables and 28-day mortality as the dependent variable. Multicollinearity among covariates included in the multivariable regression models was assessed using variance inflation factors (VIFs).

Baseline MONO, delta MONO, and slope MONO were separately evaluated because these parameters represent different aspects of immune-cell behavior, including initial immune status, cumulative change, and the rate of temporal variation, respectively.

Odds ratios (ORs) are presented with profile-likelihood 95% confidence intervals (CIs), whereas *P* values are based on Wald tests. Sequential adjustment models were constructed to evaluate the robustness of associations. Potentially relevant clinical variables, including demographic characteristics, inflammatory parameters, and conventional prognostic indicators, were considered for adjustment based on clinical relevance and exploratory findings.

To further characterize heterogeneity in MONO trajectories among patients, unsupervised clustering analysis was performed using longitudinal MONO measurements obtained during the first 7 days after admission. Partitioning around medoids (PAM), a clustering method based on patient-level trajectory similarity, was applied to identify distinct temporal patterns of MONO evolution.

The optimal number of clusters was determined based on trajectory separation and clinical interpretability. Differences in clinical characteristics and outcomes between trajectory clusters were subsequently evaluated. Kaplan–Meier survival analysis with the log-rank test was performed to compare 28-day survival distributions between clusters.

### 2.8 Exploratory prognostic modeling, interpretability analysis, and sensitivity analyses

To explore whether MONO-derived dynamic indicators provided additional prognostic information beyond conventional clinical parameters, exploratory prognostic models were developed.

Candidate predictors included conventional clinical variables and MONO-derived dynamic parameters. Model discrimination was evaluated using receiver operating characteristic (ROC) curves and the area under the curve (AUC). Given the limited sample size, leave-one-out cross-validation (LOOCV) was applied to reduce optimism bias and obtain a more conservative estimation of model performance. AUCs were reported with 95% confidence intervals estimated using 2,000 bootstrap resamples. Pairwise comparisons between ROC curves were performed using DeLong's test, with *P* values adjusted for multiple pairwise comparisons using the Holm method.

Calibration analysis was performed to assess the agreement between predicted and observed mortality probabilities. Decision curve analysis (DCA) was conducted to evaluate the potential clinical utility of models across different threshold probabilities.

To improve interpretability of the predictive models, Shapley Additive Explanations (SHAP) analysis was performed. SHAP values were calculated to quantify the contribution of individual predictors to model predictions and to identify the relative importance of MONO-derived dynamic parameters compared with conventional clinical variables.

Spearman correlation analysis was additionally performed to explore relationships between MONO-derived indicators and clinically relevant parameters, including inflammatory markers, coagulation-related variables, organ dysfunction indicators, and prognostic scores.

Sensitivity analysis was performed by re-including the patient excluded due to pre-existing bone marrow suppression and repeating the primary longitudinal and regression analyses to assess the robustness of the findings.

To address the possibility that measurements obtained later in the original 7-day observation window might reflect biological changes occurring close to death, an additional shorter-window analysis was performed using MONO measurements obtained within the first 72 h after admission. Patients were included if they had a baseline MONO measurement and at least one additional MONO measurement within 72 h. Delta MONO and slope MONO were recalculated using measurements within this restricted window. MONO trajectories were reassessed using linear mixed-effects models, and PAM clustering and Kaplan–Meier analysis were repeated using the 72 h trajectory features. Multivariable logistic regression models were constructed using the same adjustment strategy as in the primary analysis. Exploratory LOOCV-based prognostic modeling and SHAP analysis were additionally performed to assess the contribution of 72 h MONO-derived dynamic indicators.

To evaluate whether the observed association between MONO dynamics and mortality was primarily attributable to infection-related immune alterations, a sensitivity analysis was performed after excluding patients who developed sepsis during hospitalization. Longitudinal MONO trajectories and multivariable associations of MONO-derived indicators with 28-day mortality were reassessed in the non-sepsis subgroup.

Given the marked age difference between survivors and non-survivors, additional age-related sensitivity analyses were conducted. Patients were stratified according to the median age of the cohort, and MONO trajectories and associations between delta MONO and 28-day mortality were reassessed within each age stratum. Because of the limited subgroup sample sizes, Firth logistic regression was used for age-stratified analyses. A formal interaction model including age as a continuous variable and an age × delta MONO interaction term was additionally fitted to assess potential effect modification by age.

### 2.9 Statistical analysis

Continuous variables were assessed for distribution characteristics and summarized as mean ± standard deviation (SD) or median with interquartile range (IQR), as appropriate. Categorical variables were presented as frequencies and percentages.

Comparisons between survivors and non-survivors were performed using Student's t test or Mann–Whitney U test for continuous variables and chi-square test or Fisher's exact test for categorical variables, depending on data characteristics.

For longitudinal analyses, repeated measurements were analyzed using linear mixed-effects models as described above. For exploratory comparisons among multiple trajectory clusters, one-way ANOVA or Kruskal–Wallis test was used for continuous variables, and chi-square test or Fisher's exact test was applied for categorical variables.

All statistical tests were two-sided, and *P* < 0.05 was considered statistically significant. Considering the exploratory nature of this study and the limited sample size, statistical findings were interpreted cautiously.

All analyses were performed using R software (version 4.3.0) and Python software (version 3.10.6).

## 3 Results

### 3.1 Cohort identification and baseline characteristics of rigorously defined ALF patients

A total of 266 patients with ICD-coded ALF were initially identified from the hospital information system between October 2018 and January 2026. These patients were distributed across multiple clinical departments, with the largest proportions originating from the intensive care unit (ICU, *n* = 106, 39.8%) and the Department of Infectious Diseases (*n* = 60, 22.6%). The remaining cases were scattered among various medical and surgical departments, including cardiology, gastroenterology, geriatrics, oncology, and other specialties. This broad distribution indicated substantial heterogeneity among ICD-coded ALF diagnoses in real-world clinical practice.

The final cohort consisted of 20 males (43.5%) and 26 females (56.5%), with a mean age of 48.4 years. The 28-day mortality rate was 50.0% (23/46), and deaths occurred predominantly during the early phase after admission, with a median survival time of 4 days among non-survivors. After strict case verification, eligible patients were mainly derived from the Department of Infectious Diseases (*n* = 32, 69.6%), ICU (*n* = 10, 21.7%), and Gastroenterology Department (*n* = 4, 8.7%).

The etiological background of ALF was heterogeneous. Drug- or substance-related exposure was frequently identified, including conventional medications, traditional Chinese medicines, and other non-prescription products. Viral hepatitis-related ALF was relatively uncommon, with only a small proportion of patients attributable to acute HBV or HEV infection. Although a definite single cause could not be established in several retrospective cases, most patients had potential precipitating factors before disease onset.

Patients frequently presented with multisystem complications. Electrolyte disorders were the most common complication (*n* = 33, 71.7%), followed by ascites (*n* = 25, 54.3%), respiratory failure (*n* = 18, 39.1%), and renal failure (*n* = 17, 37.0%). Infection-related complications were also observed, including respiratory infection (*n* = 14, 30.4%), sepsis (*n* = 12, 26.1%), and abdominal infection (*n* = 7, 15.2%). Regarding treatment intensity, 11 patients (23.9%) required ICU admission, 13 (28.3%) received mechanical ventilation, 12 (26.1%) received vasopressor support, and 9 (19.6%) underwent continuous renal replacement therapy. Artificial liver support was performed in 28 patients (60.9%), reflecting the high severity of illness in this cohort.

### 3.2 Baseline demographic and clinical characteristics

Baseline demographic, clinical, and laboratory characteristics of survivors and non-survivors are summarized in Table 1. Compared with survivors, non-survivors were significantly older (58.3 ± 14.9 vs. 38.6 ± 15.5 years, *P* < 0.001) and more frequently required intensive care management, with a higher proportion of ICU admission (43.5% vs. 4.3%, *P* = 0.004). No significant difference was observed in sex distribution between the two groups.

**Table 1:** Baseline clinical characteristics and laboratory findings according to 28-day survival status.

Variable	Overall	Survivors	Non-survivors	*P*_value
Gender	20 (43.5%)	10 (43.5%)	10 (43.5%)	1.000
Age	48.41 ± 18.04	38.57 ± 15.48	58.26 ± 14.93	**<0** **.** **001**
Ascites	25 (54.3%)	10 (43.5%)	15 (65.2%)	0.139
Pleural effusion	11 (23.9%)	3 (13.0%)	8 (34.8%)	0.165
Sepsis	12 (26.1%)	1 (4.3%)	11 (47.8%)	**0** **.** **002**
Electrolyte disturbance	33 (71.7%)	14 (60.9%)	19 (82.6%)	0.189
Gastrointestinal bleeding	9 (19.6%)	3 (13.0%)	6 (26.1%)	0.459
Respiratory tract infection	14 (30.4%)	3 (13.0%)	11 (47.8%)	**0** **.** **023**
Abdominal infection	7 (15.2%)	0 (0.0%)	7 (30.4%)	**0** **.** **009**
Renal failure	17 (37.0%)	3 (13.0%)	14 (60.9%)	**0** **.** **002**
Respiratory failure	18 (39.1%)	2 (8.7%)	16 (69.6%)	**<0** **.** **001**
ICU	11 (23.9%)	1 (4.3%)	10 (43.5%)	**0** **.** **004**
Mechanical ventilation	13 (28.3%)	2 (8.7%)	11 (47.8%)	**0** **.** **007**
Vasopressor use	12 (26.1%)	2 (8.7%)	10 (43.5%)	**0** **.** **017**
CRRT	9 (19.6%)	1 (4.3%)	8 (34.8%)	**0** **.** **022**
Artificial liver support therapy	28 (60.9%)	14 (60.9%)	14 (60.9%)	1.000
WBC	7.90 (5.78, 12.18)	6.20 (4.90, 7.55)	10.50 (8.25, 15.25)	**<0** **.** **001**
RBC	4.41 ± 0.82	4.56 ± 0.75	4.27 ± 0.88	0.250
HGB	133.14 ± 21.95	136.32 ± 19.66	129.96 ± 24.04	0.331
HCT	38.93 ± 7.01	39.69 ± 6.18	38.17 ± 7.81	0.468
MCV	89.81 ± 8.42	88.80 ± 9.08	90.82 ± 7.76	0.422
MCH	30.37 ± 3.35	30.15 ± 4.00	30.59 ± 2.60	0.665
MCHC	339.50 (329.25, 347.00)	341.00 (329.50, 347.00)	339.00 (330.00, 347.00)	0.817
RDW	15.35 (13.83, 17.70)	14.50 (13.45, 17.10)	15.90 (14.60, 18.10)	**0** **.** **050**
SD	47.40 (41.78, 54.00)	43.20 (40.45, 50.05)	50.10 (43.70, 56.35)	0.141
PLT	135.50 (90.75, 197.50)	142.00 (111.00, 207.00)	115.00 (63.00, 184.00)	0.063
PDW	15.05 ± 2.70	14.78 ± 2.39	15.33 ± 3.02	0.495
PLT-PCT	0.16 ± 0.08	0.18 ± 0.08	0.13 ± 0.06	**0** **.** **019**
MPV	11.11 ± 1.26	11.05 ± 1.03	11.17 ± 1.47	0.747
LYMPH	1.15 (0.71, 1.66)	1.38 (0.90, 1.64)	0.98 (0.66, 1.75)	0.606
MONO	0.70 (0.50, 0.90)	0.69 (0.50, 0.84)	0.73 (0.55, 1.14)	0.385
NEUT	5.39 (3.76, 9.55)	3.80 (2.66, 5.83)	7.81 (5.27, 11.20)	**0** **.** **001**
EO	0.10 (0.02, 0.14)	0.09 (0.02, 0.14)	0.10 (0.02, 0.13)	0.930
NLR	5.57 (2.43, 10.89)	3.62 (1.69, 6.98)	7.41 (4.54, 14.59)	**0** **.** **019**
MLR	0.58 (0.35, 1.07)	0.49 (0.32, 0.80)	0.74 (0.47, 1.23)	0.210
PLR	119.16 (76.92, 201.98)	131.91 (87.31, 225.64)	108.29 (69.77, 192.03)	0.345
SII	748.27 (390.50, 1,432.90)	583.33 (275.43, 1,371.07)	858.27 (493.26, 1,421.36)	0.187
SIRI	3.97 (1.29, 7.89)	2.31 (0.96, 6.47)	5.62 (3.28, 9.20)	**0** **.** **025**
PT-S	29.10 (22.47, 46.38)	26.80 (22.30, 30.40)	37.30 (25.40, 58.40)	0.052
PT%	29.00 (18.35, 36.95)	33.00 (29.00, 38.00)	20.00 (12.70, 28.20)	**0** **.** **007**
INR	2.61 (1.96, 3.46)	2.14 (1.93, 2.61)	3.07 (2.37, 4.71)	**0** **.** **026**
APTT	48.04 ± 15.23	41.51 ± 10.64	54.57 ± 16.49	**0** **.** **003**
TT	20.71 ± 4.99	19.92 ± 4.86	21.50 ± 5.11	0.287
AT-III	29.25 (17.23, 61.05)	38.00 (22.00, 63.50)	22.30 (14.45, 54.10)	0.104
FDP	13.07 (2.24, 32.39)	9.29 (1.07, 27.63)	14.09 (5.73, 39.80)	0.187
D-D	227.33 (5.85, 1,670.75)	295.00 (2.28, 1,429.50)	214.67 (5.96, 1,319.50)	0.956
FIB-C	1.31 (0.94, 1.92)	1.44 (1.25, 2.00)	1.16 (0.74, 1.66)	0.053
ALT	1,140.00 (583.50, 2,369.25)	1,394.00 (707.35, 3,169.00)	845.49 (276.50, 2,077.00)	0.062
AST	1,025.50 (365.25, 2,125.50)	1,017.00 (565.50, 2,541.57)	1,034.00 (280.50, 1,686.95)	0.282
TP	59.00 ± 9.66	61.30 ± 7.43	56.69 ± 11.16	0.107
ALB	33.78 ± 5.85	35.08 ± 5.31	32.47 ± 6.19	0.133
GLB	25.43 ± 6.50	26.82 ± 4.51	24.04 ± 7.87	0.151
TBIL	235.01 (122.92, 414.50)	193.10 (121.40, 391.15)	288.10 (125.05, 563.20)	0.538
DBIL	130.85 (67.50, 285.80)	130.30 (76.30, 230.50)	150.50 (60.60, 332.50)	0.709
IBIL	94.55 (35.90, 192.90)	84.00 (39.45, 131.10)	109.20 (35.20, 197.25)	0.429
CHE	3,958.50 (3,067.50, 6,132.50)	4,270.00 (3,130.00, 5,840.00)	3,780.00 (3,085.00, 6,480.00)	0.843
ALP	149.50 (115.25, 188.50)	157.05 (129.50, 178.00)	136.00 (112.00, 226.00)	1.000
GGT	115.31 (52.25, 161.00)	145.00 (67.50, 184.00)	84.00 (48.50, 152.00)	0.163
TBA	217.00 (110.34, 289.00)	190.60 (119.72, 305.50)	251.50 (76.70, 289.00)	0.667
GLU	5.92 (3.90, 8.01)	5.36 (3.93, 6.67)	6.86 (3.51, 11.27)	0.227
TG	2.82 (2.10, 4.08)	2.92 (2.52, 4.08)	2.61 (1.69, 4.08)	0.266
TC	1.31 (0.76, 2.30)	1.36 (0.90, 2.01)	1.26 (0.68, 2.50)	0.741
HDL	0.62 (0.43, 0.90)	0.63 (0.52, 0.76)	0.48 (0.35, 0.90)	0.441
LDL	1.63 (1.08, 2.43)	1.55 (1.31, 2.40)	1.71 (0.93, 2.50)	0.991
K	3.85 (3.47, 4.33)	3.71 (3.49, 4.14)	4.11 (3.39, 4.44)	0.442
Na	137.72 ± 4.92	138.09 ± 3.38	137.35 ± 6.15	0.617
Cl	101.21 ± 7.00	101.95 ± 5.71	100.47 ± 8.15	0.479
Urea	4.10 (2.63, 8.42)	3.62 (2.68, 5.33)	4.44 (2.88, 8.85)	0.187
CRE	66.00 (47.25, 96.68)	55.00 (47.00, 77.00)	85.00 (51.25, 105.25)	0.116
PCT	1.00 (0.53, 2.98)	1.05 (0.75, 3.31)	0.95 (0.38, 1.70)	0.286

Bold values indicate statistical significance (*P* < 0.05).

Non-survivors exhibited a greater burden of infection-related complications and organ dysfunction, including higher frequencies of sepsis, respiratory infection, abdominal infection, renal failure, and respiratory failure. Consistently, more intensive organ support therapies were required among non-survivors, including CRRT (34.8% vs. 4.3%), mechanical ventilation (47.8% vs. 8.7%), and vasopressor support (43.5% vs. 8.7%). The proportion of patients receiving artificial liver support was comparable between groups (60.9% in both groups).

Baseline laboratory analyses revealed more pronounced inflammatory activation and coagulation abnormalities among non-survivors. Non-survivors had significantly higher white blood cell counts [10.50 (8.25–15.25) vs. 6.20 (4.90–7.55), *P* < 0.001] and neutrophil counts [7.81 (5.27–11.20) vs. 3.80 (2.66–5.83), *P* = 0.001], whereas plateletcrit was significantly lower (0.13 ± 0.06% vs. 0.18 ± 0.08%, *P* = 0.019). Other hematological parameters, including baseline MONO, lymphocyte counts, platelet counts, hemoglobin levels, and eosinophil counts, showed no significant differences between survivors and non-survivors.

Coagulation dysfunction was more severe in non-survivors, characterized by lower PTA [20.00 (12.70–28.20)% vs. 33.00 (29.00–38.00), *P* = 0.007], higher INR [3.07 (2.37–4.71) vs. 2.14 (1.93–2.61), *P* = 0.026], and prolonged APTT (54.57 ± 16.49 vs. 41.51 ± 10.64, *P* = 0.003). In contrast, most biochemical indicators, including aminotransferases, bilirubin, albumin, renal function parameters, and procalcitonin, did not significantly differ between groups.

Among inflammation-related composite indices, NLR [7.41 (4.54–14.59) vs. 3.62 (1.69–6.98), *P* = 0.019] and SIRI [5.62 (3.28–9.20) vs. 2.31 (0.96–6.47), *P* = 0.025] were significantly elevated in non-survivors, whereas MLR, PLR, and SII showed no significant differences.

### 3.3 Longitudinal changes in peripheral blood cell parameters during the first 7 days

To determine whether early immune-cell dynamics differed according to clinical outcome, longitudinal trajectories of peripheral blood cell parameters were compared between survivors and non-survivors during the first 7 days after admission. All included patients contributed at least two longitudinal measurements during the observation period, allowing assessment of individual temporal changes rather than relying solely on baseline values.

**Figure 2:**
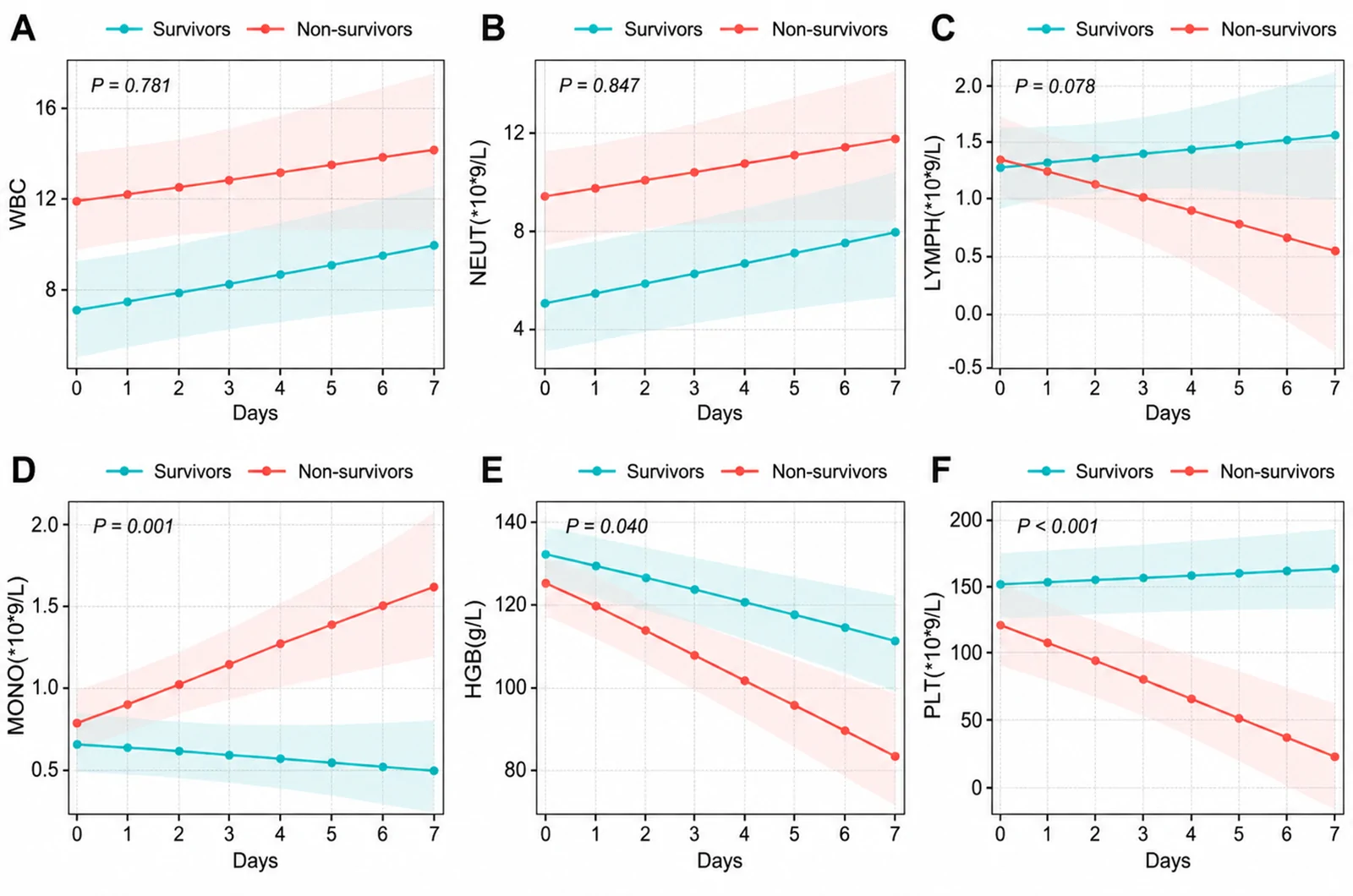
Longitudinal trajectories of peripheral blood cell parameters during the first 7 days after admission. Trajectories are shown for survivors (*n* = 23) and non-survivors (*n* = 23). **(A)** WBC. **(B)** NEUT. **(C)** LYMPH. **(D)** MONO. **(E)** HGB. **(F)** PLT. Differences in temporal trajectories between survivors and non-survivors were evaluated using linear mixed-effects models accounting for repeated measurements within individuals. The reported *P* values represent the time × survival-group interaction.

As shown in Figure 2, WBC and NEUT remained consistently higher in non-survivors throughout the observation period, suggesting sustained inflammatory activation in patients with poor outcomes. However, neither WBC nor NEUT demonstrated significant differences in temporal evolution between groups, with no significant group-by-time interaction observed (WBC, *P* = 0.781; NEUT, *P* = 0.847). LYMPH showed a trend toward divergent trajectories between survivors and non-survivors, although the interaction did not reach statistical significance (*P* = 0.078).

In contrast, MONO demonstrated a distinct and progressive divergence between the two outcome groups. MONO levels remained relatively stable or showed a slight decline among survivors, whereas they progressively increased during the first 7 days after admission among non-survivors. This temporal separation resulted in a significant group-by-time interaction (*P* = 0.001), indicating substantially different MONO evolution patterns according to survival status. Importantly, this difference was not evident from baseline MONO measurements alone but emerged during serial follow-up, suggesting that longitudinal MONO dynamics may capture clinically relevant immune alterations during ALF progression.

Among other blood cell parameters, hemoglobin levels declined in both groups but showed a more pronounced decrease among non-survivors (*P* = 0.040). Platelet counts demonstrated the most prominent overall divergence, with relative stability or mild elevation among survivors and progressive decline among non-survivors (*P* < 0.001). These findings indicate that early temporal changes in peripheral blood cell populations differ according to clinical outcome, with MONO trajectories showing the most consistent separation between survivors and non-survivors.

Additional exploratory longitudinal analyses of inflammatory indices, coagulation parameters, and liver function-related markers showed limited temporal differences between groups. Among inflammatory indices, only MLR showed a borderline difference (*P* = 0.073), whereas NLR, PLR, SII, and SIRI did not demonstrate significant trajectory differences. For coagulation parameters, PTA showed a more favorable increase among survivors (*P* = 0.030), while prothrombin time, INR, and APTT showed no significant group-specific temporal patterns (Supplementary Figures S1–S3).

To formally evaluate the difference in MONO trajectories while accounting for repeated measurements within individuals, linear mixed-effects models were subsequently applied (Table 2). After adjustment for potential confounders, including age, gender, and baseline WBC, the group-by-time interaction for MONO remained statistically significant across all models (Model 1: *β* = 0.006, 95% CI: 0.002–0.010, *P* = 0.001; Model 2: *β* = 0.006, 95% CI: 0.002–0.010, *P* = 0.001; Model 3: *β* = 0.006, 95% CI: 0.003–0.010, *P* < 0.001), supporting the robustness of the observed difference in MONO trajectories after adjustment for age, gender, and baseline WBC.

**Table 2:** Linear mixed-effects models for early MONO trajectories during the first 7 days after admission.

Variable	Model 1 (95%CI)	*p*-value	Model 2 (95%CI)	*p*-value	Model 3 (95%CI)	*p*-value
Intercept	0.686 (0.490, 0.883)	**<0** **.** **001**	0.788 (0.414, 1.161)	**<0**.**001**	0.647 (0.304, 0.990)	**<0**.**001**
Day	−0.001 (−0.003, 0.001)	0.412	−0.001 (−0.003, 0.001)	0.431	−0.002 (−0.003, 0.000)	0.058
Outcome	0.149 (−0.129, 0.427)	0.289	0.243 (−0.075, 0.561)	0.132	0.075 (−0.219, 0.369)	0.612
Day × Outcome	0.006 (0.002, 0.010)	**0**.**001**	0.006 (0.002, 0.010)	**0**.**001**	0.006 (0.003, 0.010)	**<0**.**001**

Model 1: Age.

Model 2: Age + Gender.

Model 3: Age + Gender + WBC.

Bold values indicate statistical significance (*P* < 0.05).

### 3.4 Patient-level MONO dynamics: individual trajectories, delta MONO, and slope MONO

Following the observation of distinct population-level MONO trajectories between survivors and non-survivors, we further evaluated individual-level MONO dynamics to characterize the heterogeneity of temporal immune responses among patients.

Patient-level trajectory analysis demonstrated substantial interindividual variability in MONO evolution during the first 7 days after admission (Supplementary Figure S4). Although baseline MONO levels were comparable between survivors and non-survivors, their subsequent temporal patterns differed markedly. Most survivors exhibited relatively stable or gradually declining MONO trajectories, whereas several non-survivors showed progressive increases in MONO during early disease progression.

To quantitatively characterize these dynamic changes, two patient-level MONO-derived indicators were calculated: delta MONO, representing the magnitude of MONO change during the observation period, and slope MONO, reflecting the average rate of MONO variation over time. Both delta MONO and slope MONO were significantly higher in non-survivors than in survivors (both *P* < 0.001), indicating greater absolute and rate-based MONO changes among patients with poor outcomes at the descriptive level.

These findings further supported the concept that dynamic MONO changes, rather than baseline MONO values alone, may better reflect clinically relevant immune alterations associated with ALF progression.

### 3.5 Exploratory MONO trajectory clustering and survival analysis

To further investigate whether distinct patterns of early MONO evolution represented clinically meaningful phenotypes, unsupervised trajectory clustering was performed using serial MONO measurements obtained during the first 7 days after admission.

**Figure 3:**
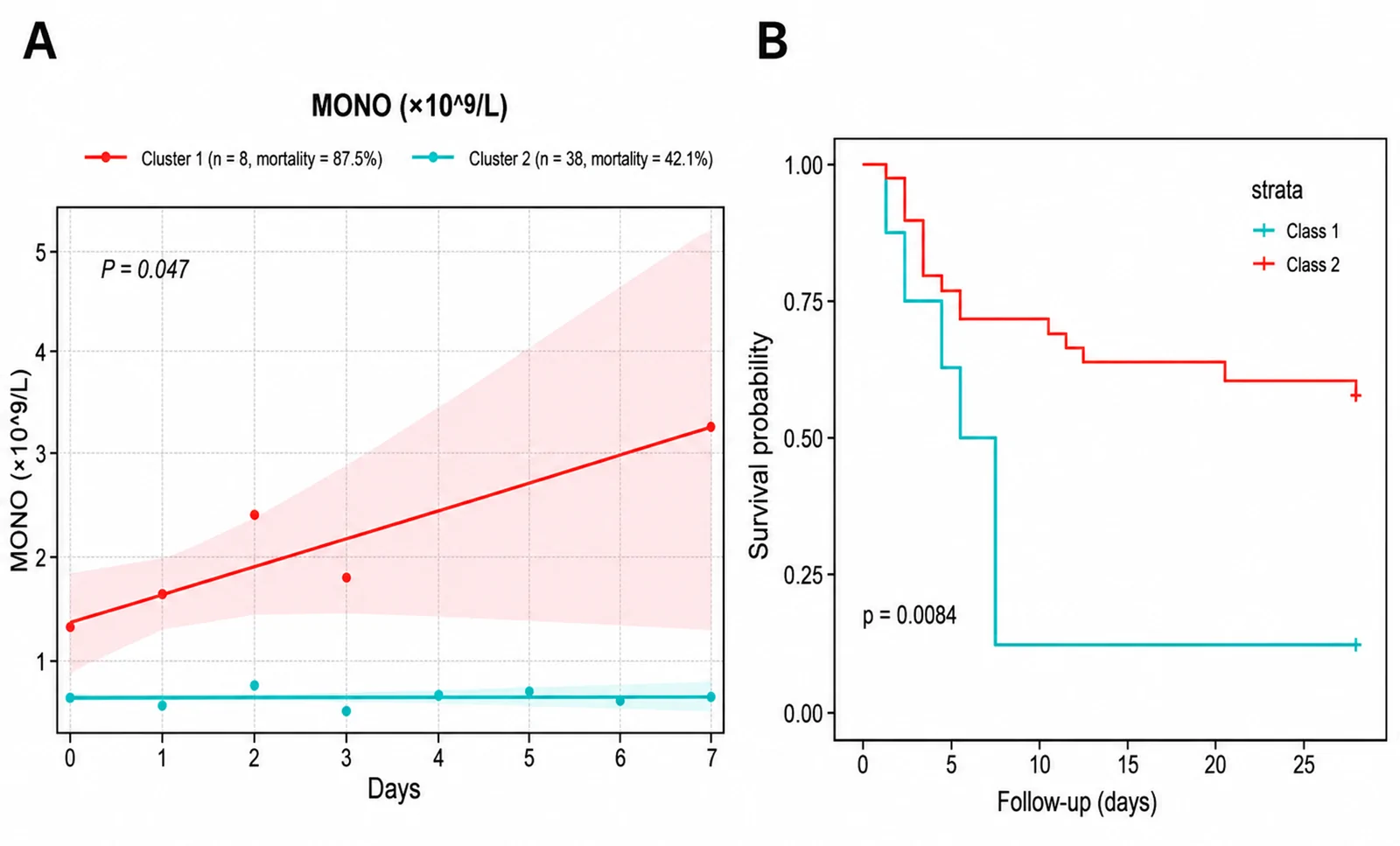
Exploratory MONO trajectory-based clustering and 28-day survival analysis. A total of 46 patients were included in the trajectory-clustering analysis. **(A)** PAM clustering of longitudinal peripheral monocyte (MONO) measurements obtained during the first 7 days after admission identified two trajectory phenotypes: Cluster 1 (*n* = 8), characterized by a progressive increase in MONO, and Cluster 2 (*n* = 38), characterized by a relatively stable MONO trajectory. **(B)** Kaplan–Meier curves comparing 28-day survival between the two PAM-derived clusters. Survival distributions were compared using the log-rank test.

Partitioning around medoids clustering identified two major MONO trajectory patterns. Cluster 1 (*n* = 8) was characterized by a progressive increase in MONO levels over time, whereas Cluster 2 (*n* = 38) showed relatively stable MONO trajectories (Figure 3). Patients in Cluster 1 exhibited significantly higher 28-day mortality compared with those in Cluster 2 (87.5% vs. 42.1%, *P* = 0.047). Consistently, Kaplan–Meier survival analysis demonstrated significantly poorer 28-day survival in Cluster 1 (log-rank *P* = 0.0084), suggesting that MONO trajectory patterns were associated with clinically relevant outcome differences.

Baseline characteristics of the two trajectory clusters are summarized in Supplementary Table S1. No significant differences were observed in age, sex, ICU admission, or major organ support therapies between clusters. However, patients in Cluster 1 showed higher baseline inflammatory activity, characterized by increased baseline WBC, MONO, and NEUT counts, as well as a higher frequency of respiratory infection and respiratory failure.

Laboratory analyses further indicated that Cluster 1 was associated with greater systemic disturbance, including more severe coagulation abnormalities (prolonged APTT and lower fibrinogen levels) and increased inflammatory burden reflected by elevated MLR and SIRI.

To determine whether trajectory-based clustering captured a MONO-specific prognostic pattern, exploratory clustering analyses were additionally performed for other clinical parameters. Most alternative parameters showed less consistent trajectory separation, whereas PTA demonstrated relatively clearer discrimination between outcome groups (Supplementary Figure S5). These findings suggest that dynamic MONO patterns may represent a clinically relevant immune trajectory associated with short-term mortality in ALF.

### 3.6 Associations of MONO-derived indicators with 28-day mortality

Multivariable logistic regression analyses were performed to further evaluate the associations between baseline MONO and MONO-derived dynamic indicators with 28-day mortality (Table 3). No substantial multicollinearity was detected among covariates included in the multivariable models; all VIF values were <5, with the maximum observed VIF being 3.135.

**Table 3:** Multivariable logistic regression analyses of MONO-derived indicators and exploratory trajectory-informed clusters for 28-day mortality.

Exposure	Model 1	Model 2	Model 3A	Model 3B	Model 3C	Model 3D	Model 3E	Model 3F
MONO	1.120 (0.973, 1.334), *P* = 0.148	1.260 (1.024, 1.682), *P* = 0.065	1.198 (0.955, 1.669), *P* = 0.194	1.370 (1.047, 1.982), *P* = 0.053	1.242 (0.920, 1.809), *P* = 0.202	1.356 (1.017, 2.006), *P* = 0.084	1.720 (1.203, 2.838), *P* = 0.010	1.318 (0.984, 1.914), *P* = 0.095
Delta MONO	1.212 (1.058, 1.453), ***P* = 0.016**	1.273 (1.075, 1.617), ***P* = 0.016**	1.324 (1.072, 1.851), ***P* = 0.035**	1.257 (1.042, 1.658), ***P* = 0.045**	1.263 (1.034, 1.726), ***P* = 0.043**	1.373 (1.101, 1.978), ***P* = 0.024**	1.330 (1.083, 1.852), ***P* = 0.028**	1.351 (1.078, 1.964), ***P* = 0.033**
Slope MONO	1.042 (0.998, 1.107), *P* = 0.105	1.047 (0.999, 1.113), *P* = 0.077	1.049 (0.990, 1.140), *P* = 0.159	1.033 (0.980, 1.105), *P* = 0.268	1.035 (0.983, 1.112), *P* = 0.226	1.056 (0.998, 1.146), *P* = 0.098	1.044 (0.991, 1.119), *P* = 0.132	1.048 (0.992, 1.129), *P* = 0.107
Cluster 2 vs. Cluster 1	0.104 (0.005, 0.665), ***P* = 0.043**	0.023 (0.001, 0.288), ***P* = 0.011**	0.020 (0.000, 0.404), ***P* = 0.025**	0.018 (0.000, 0.303), ***P* = 0.015**	0.024 (0.001, 0.440), ***P* = 0.022**	0.002 (0.000, 0.089), ***P* = 0.006**	0.002 (0.000, 0.066), ***P* = 0.004**	0.008 (0.000, 0.178), ***P* = 0.007**

Model 1: Raw model.

Model 2: Age + Gender.

Model 3A: Age + Gender + WBC + MELD.

Model 3B: Age + Gender + NLR + MELD.

Model 3C: Age + Gender + Sepsis + MELD.

Model 3D: Age + Gender + WBC + PTA.

Model 3E: Age + Gender + NLR + PTA.

Model 3F: Age + Gender + Sepsis + PTA.

Bold values indicate statistical significance (*P* < 0.05).

Baseline MONO showed a relatively weak and model-dependent association with mortality. Although statistical significance was observed in the fully adjusted model (OR = 1.720, 95% CI: 1.203–2.838, *P* = 0.010), this association was not consistently observed across different adjustment models. In contrast, delta MONO showed the most consistent association with 28-day mortality. Higher delta MONO was associated with increased mortality risk in the unadjusted model (OR = 1.212, 95% CI: 1.058–1.453, *P* = 0.016), and this association remained significant after sequential adjustment for demographic characteristics and clinically relevant prognostic variables, including age, gender, WBC, NLR, MELD, and PTA (OR range: 1.257–1.373).

Slope MONO showed a positive association with mortality across regression models, although statistical significance was not consistently achieved. In comparison, trajectory-based MONO clustering demonstrated a stronger and more stable association with outcome. Compared with patients in Cluster 1 characterized by progressive MONO elevation, patients in Cluster 2 with relatively stable MONO trajectories showed significantly lower odds of 28-day mortality across all adjusted models.

Full regression coefficients for all covariates included in the primary multivariable models are provided in Supplementary Table S2. The directions of the associations for conventional severity-related covariates were generally consistent across models, with higher MELD scores tending to be associated with increased mortality risk and higher PTA tending to be associated with lower mortality risk.

To further explore the clinical relevance of MONO dynamics, patients were stratified according to delta MONO and slope MONO. Patients with delta MONO > 0 had significantly higher 28-day mortality than those with delta MONO ≤ 0 (65.5% vs. 23.5%, *P* = 0.006). These patients also showed higher frequencies of ICU admission, mechanical ventilation, vasopressor support, and CRRT, together with increased rates of renal and respiratory failure. However, baseline MELD score, MELD-Na score, KCC positivity, and baseline laboratory parameters were comparable between the two groups (Supplementary Table S3).

When patients were categorized according to slope MONO tertiles, a progressive increase in 28-day mortality was observed across tertiles (22.7%, 63.6%, and 84.6%, respectively; *P* = 0.001). Higher slope MONO tertiles were also associated with increased ICU admission, organ support requirements, and organ dysfunction. In contrast, conventional prognostic scores, including MELD, MELD-Na, and KCC, did not demonstrate significant differences across slope MONO tertile groups (Supplementary Table S4).

### 3.7 Exploratory assessment of complementary prognostic information

To investigate whether MONO-derived dynamic indicators provided additional prognostic information beyond conventional clinical variables, exploratory prognostic models were developed and evaluated using leave-one-out cross-validation.

Four models were constructed: a baseline clinical model, a PTA-containing model, a MONO dynamic model incorporating MONO-derived indicators, and a combined model integrating both PTA and MONO dynamic parameters.

The baseline model achieved an AUC of 0.784 (95% CI: 0.640–0.905), whereas the model incorporating PTA alone showed an AUC of 0.762 (95% CI: 0.606–0.897). In comparison, the MONO dynamic model showed a numerically higher AUC (AUC = 0.858) (95% CI: 0.737–0.956). The combined model including both PTA and MONO-derived indicators showed the highest observed AUC (AUC = 0.875) (95% CI: 0.758–0.963), suggesting that dynamic MONO parameters may provide complementary prognostic information beyond conventional clinical variables.

**Figure 4:**
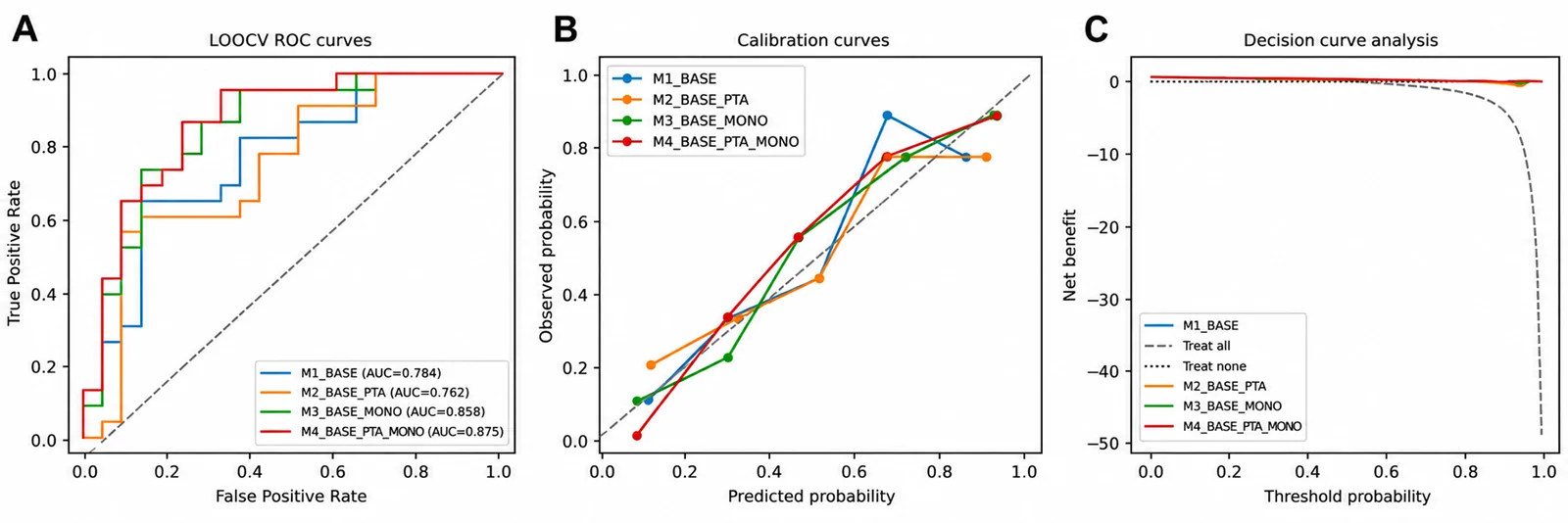
Exploratory performance of prognostic models incorporating MONO-derived dynamic indicators for 28-day mortality. Analyses were performed in the full cohort (*n* = 46). Four exploratory prognostic models were evaluated using LOOCV: a baseline clinical model (M1), a baseline model incorporating PTA (M2), a model incorporating MONO-derived dynamic indicators (M3), and a combined model incorporating both PTA and MONO-derived indicators (M4). **(A)** LOOCV ROC with corresponding AUCs. **(B)** Calibration curves comparing predicted and observed 28-day mortality probabilities. **(C)** Decision curve analysis showing the net benefit of each model across a range of threshold probabilities; “treat all” and “treat none” are shown as reference strategies.

Calibration analysis showed acceptable agreement between predicted and observed mortality probabilities across models, although some variability was observed, likely reflecting the limited sample size. Decision curve analysis suggested a numerically higher potential clinical net benefit for models incorporating MONO-derived information across part of the threshold probability range (Figure 4).

Pairwise DeLong comparison demonstrated that the combined model achieved significantly higher discrimination than the PTA model before correction (AUC: 0.875 vs. 0.762, *P* = 0.023). However, none of the pairwise comparisons remained statistically significant after Holm correction (Supplementary Table S5).

### 3.8 Exploratory SHAP analysis

To further interpret the contribution of individual predictors within the prognostic model, exploratory SHAP analysis was performed.

**Figure 5:**
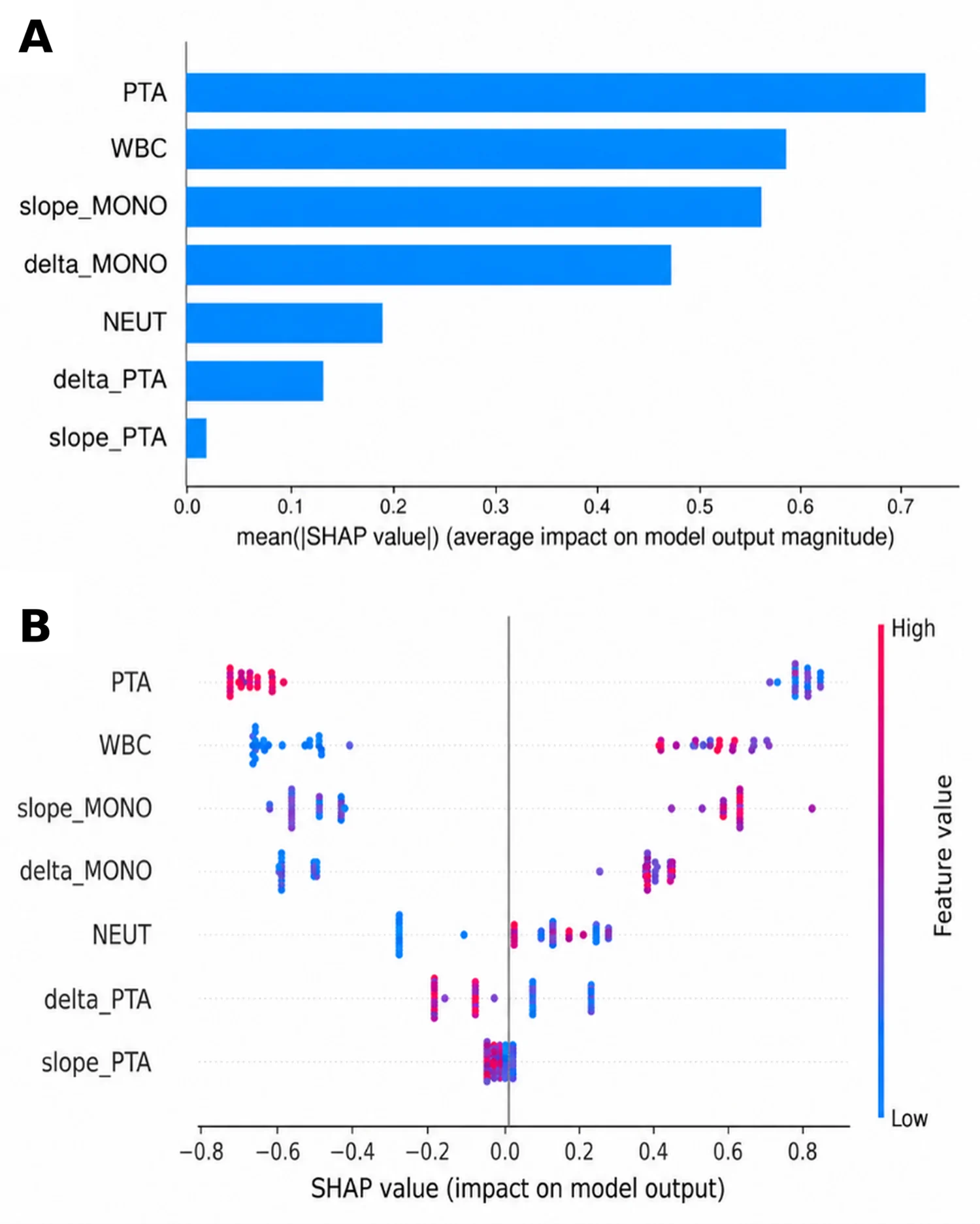
Exploratory SHAP analysis of the combined prognostic model for 28-day mortality. SHAP analysis was performed in the full cohort (*n* = 46) to interpret the contribution of individual predictors in the combined model incorporating conventional clinical variables and dynamic MONO-related parameters. **(A)** Global feature importance ranked according to the mean absolute SHAP value, representing the average magnitude of each variable's contribution to model output. **(B)** SHAP summary plot showing the distribution of individual SHAP values for each predictor. Each point represents one patient; positive SHAP values indicate contributions toward a higher predicted probability of 28-day mortality, whereas negative values indicate contributions toward a lower predicted probability. Point color represents the corresponding feature value, ranging from low to high.

As shown in Figure 5, PTA demonstrated the highest mean absolute SHAP value, followed by WBC, slope MONO, delta MONO, NEUT, delta PTA, and slope PTA. These findings indicated that both conventional clinical parameters and MONO-derived dynamic indicators contributed to mortality prediction.

SHAP summary plots demonstrated that higher PTA values were associated with lower predicted mortality risk, whereas increased WBC, slope MONO, and delta MONO were associated with higher predicted mortality risk. NEUT showed a similar but weaker contribution pattern.

Among dynamic parameters, slope MONO and delta MONO showed greater contributions to model output than slope PTA and delta PTA, suggesting that temporal changes in MONO may provide additional prognostic information beyond dynamic platelet-related parameters.

### 3.9 Additional prognostic value of monocyte dynamics beyond conventional liver failure scores

Conventional prognostic scores were significantly associated with 28-day mortality. Compared with survivors, non-survivors had a higher proportion of KCC positivity (56.5% vs. 17.4%, *P* = 0.013) and higher MELD and MELD-Na scores [38.00 (29.50–39.00) vs. 25.00 (23.00–32.00), *P* = 0.004; 38.00 (30.00–39.00) vs. 26.00 (23.00–32.50), *P* = 0.003], indicating more severe baseline hepatic dysfunction (Table 4).

**Table 4:** Conventional prognostic criteria and scores according to 28-day survival status.

Variable	Overall	Survivors	Non-survivors	*P*_value
KCC	17 (37.0%)	4 (17.4%)	13 (56.5%)	**0** **.** **013**
MELD	30.50 (23.00, 37.75)	25.00 (23.00, 32.00)	38.00 (29.50, 39.00)	**0** **.** **004**
MELD-Na	31.00 (23.50, 37.75)	26.00 (23.00, 32.50)	38.00 (30.00, 39.00)	**0** **.** **003**

Bold values indicate statistical significance (*P* < 0.05).

**Figure 6:**
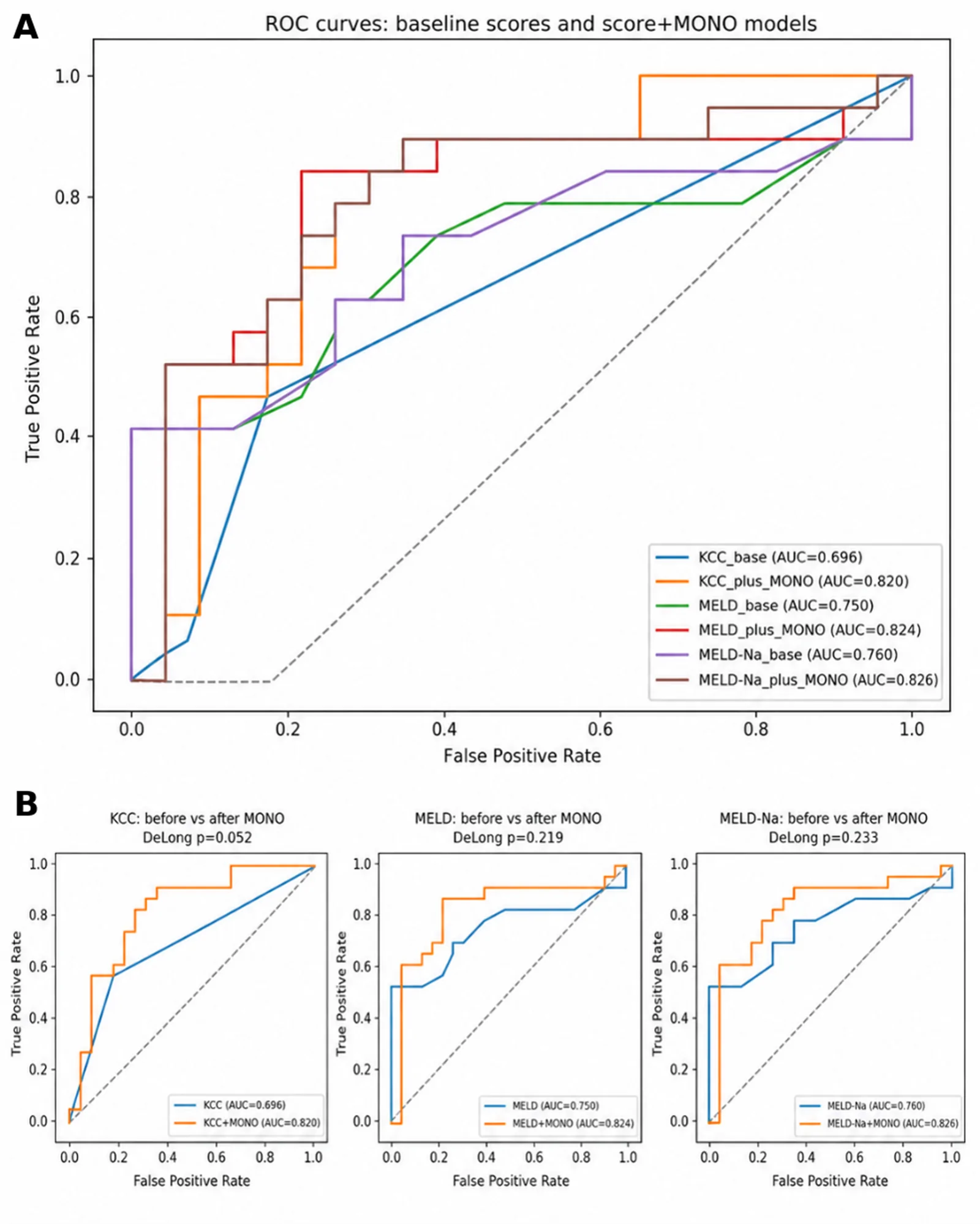
Incremental prognostic value of MONO-derived dynamic indicators beyond conventional liver failure scores. Analyses were performed in the full cohort (*n* = 46). **(A)** ROC curves comparing the discrimination of conventional prognostic scores, including KCC, MELD, and MELD-Na, with corresponding models incorporating MONO-derived dynamic indicators. AUCs are shown in the figure. **(B)** Pairwise ROC comparisons between each conventional prognostic score and its corresponding model after incorporation of MONO-derived dynamic indicators. Differences in AUCs were evaluated using DeLong's test; the displayed *P* values represent the unadjusted pairwise DeLong comparisons.

To explore whether MONO-derived dynamic information provided additional prognostic value beyond established ALF scores, MONO-related variables were incorporated into models based on KCC, MELD, and MELD-Na. The addition of MONO-derived parameters was associated with numerically higher AUCs across all three conventional models. The AUC increased from 0.696 (95% CI: 0.565–0.818) to 0.820 (95% CI: 0.686–0.933) for KCC, from 0.750 (95% CI: 0.598–0.891) to 0.824 (95% CI 0.684–0.945) for MELD, and from 0.760 (95% CI 0.611–0.896) to 0.826 (95% CI: 0.688–0.941) for MELD-Na (Figure 6).

These findings suggest that early MONO dynamics may provide complementary prognostic information beyond conventional assessments of liver dysfunction.

### 3.10 Shorter-window analysis of MONO dynamics within the first 72 h after admission

**Figure 7:**
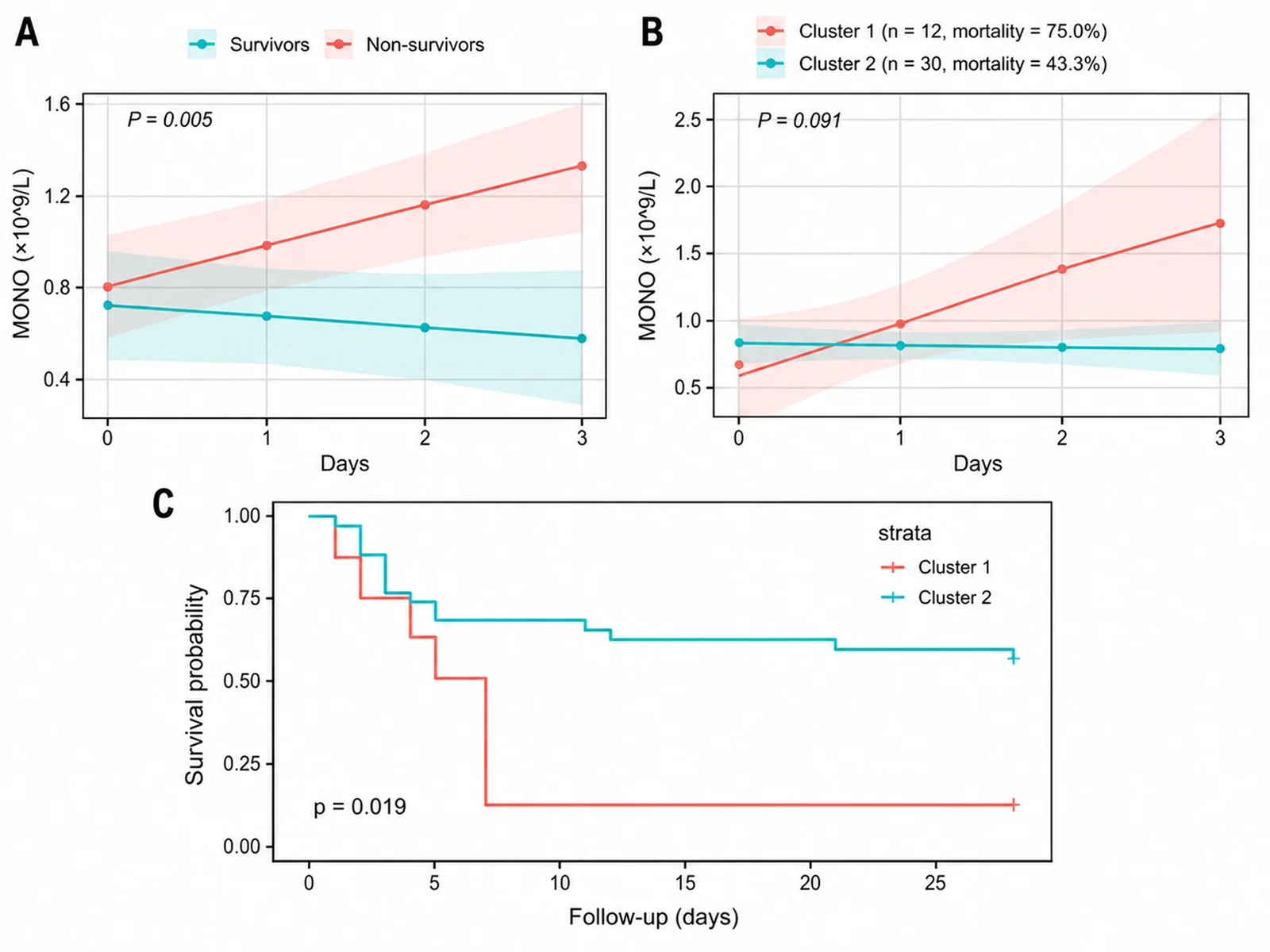
Early 72 h peripheral monocyte dynamics and 28-day outcomes in acute liver failure. A total of 42 patients with a baseline MONO measurement and at least one additional measurement within 72 h were included in this analysis, comprising 20 survivors and 22 non-survivors. **(A)** Estimated peripheral MONO trajectories during the first 72 h after admission in survivors and non-survivors based on linear mixed-effects modeling. The displayed *P* value represents the time × survival-group interaction. **(B)** PAM-derived MONO trajectory clusters within the first 72 h after admission. Cluster 1 (*n* = 12) was characterized by an increasing MONO pattern, whereas Cluster 2 (*n* = 30) showed a relatively stable trajectory. The displayed *P* value represents the between-cluster comparison of 28-day mortality using Fisher's exact test. **(C)** Kaplan–Meier curves comparing 28-day survival between the two 72 h PAM-derived trajectory clusters. Survival distributions were compared using the log-rank test.

To address the concern that MONO changes observed over the original 7-day window might partly reflect biological alterations occurring close to death, we performed an additional shorter-window analysis restricted to the first 72 h after admission. A total of 42 patients had baseline MONO measurements and at least one additional measurement within 72 h and were included in this analysis. Mixed-effects modeling demonstrated significantly different MONO trajectories between survivors and non-survivors within this early window (time × outcome interaction, *P* = 0.005), with MONO increasing over time among non-survivors but remaining stable or decreasing among survivors (Figure 7A and Supplementary Table S6).

Patient-level MONO dynamics within 72 h were further summarized using delta MONO and slope MONO. Higher delta MONO remained consistently associated with 28-day mortality across the same multivariable adjustment models used in the primary analysis, with effect estimates remaining in the same direction after adjustment for demographic characteristics, inflammatory indices, liver disease severity, and sepsis-related covariates (Supplementary Table S7). In contrast, associations for slope MONO were less consistent. Patients with delta MONO > 0 had a markedly higher 28-day mortality than those with delta MONO ≤ 0 (73.9% vs. 26.3%, *P* = 0.002), whereas MELD and MELD-Na scores were similar between the two groups (Supplementary Table S8).

PAM clustering identified two 72 h MONO trajectory phenotypes. Cluster 1 (*n* = 12) showed an increasing MONO pattern and had a numerically higher 28-day mortality than Cluster 2 (75.0% vs. 43.3%, *P* = 0.091) (Figure 7B). The corresponding trajectory-defined groups also showed different 28-day survival patterns in Kaplan–Meier analysis (log-rank *P* = 0.019) (Figure 7C). In an exploratory machine-learning analysis, the observed LOOCV AUC was 0.643 (95% CI: 0.465–0.801) for the baseline model and 0.805 (95% CI: 0.654–0.931) after incorporating 72 h delta MONO and slope MONO. SHAP analysis further indicated that delta MONO ranked among the more influential contributors to model output, whereas the relative contribution of slope MONO was smaller (Supplementary Figure S6). Collectively, these findings indicate that outcome-associated MONO divergence was already detectable within the first 72 h after admission, supporting that the primary association was not solely driven by measurements obtained during the later portion of the original 7-day observation window.

### 3.11 Exploratory SHAP analysis for organ support-related outcomespt

Exploratory analyses were performed to investigate whether MONO-derived dynamic indicators were associated with major organ support requirements, including CRRT, mechanical ventilation, and vasopressor use (Supplementary Figure S7).

Across these exploratory models, delta MONO and slope MONO showed measurable contributions to model output for different organ support outcomes. In particular, both MONO-derived indicators ranked among informative variables for mechanical ventilation and CRRT-related outcomes, suggesting a potential association between early MONO elevation and clinical deterioration requiring organ support.

### 3.12 Correlation of monocyte-derived dynamic indicators with clinical parameters

**Figure 8:**
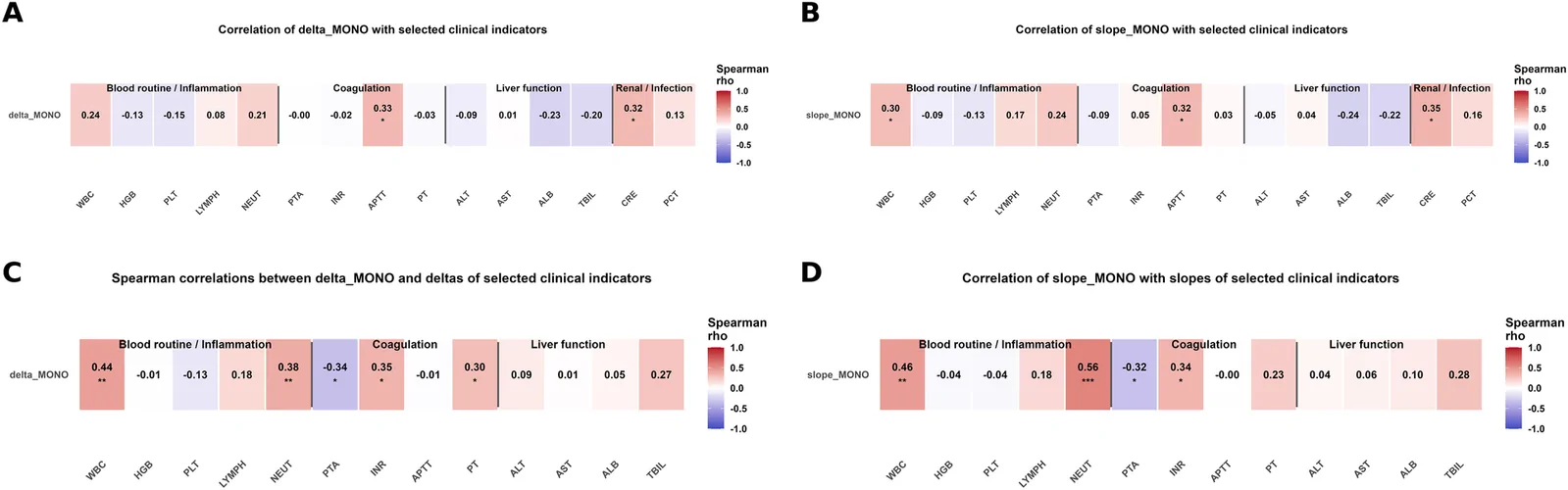
Correlation analysis of MONO-derived dynamic indicators with clinical parameters. Correlations were evaluated using Spearman rank correlation analysis in the full cohort (*n* = 46). **(A)** Correlations between delta MONO and baseline clinical parameters. **(B)** Correlations between slope MONO and baseline clinical parameters. **(C)** Correlations between delta MONO and longitudinal changes in clinical parameters. **(D)** Correlations between slope MONO and longitudinal changes in clinical parameters. Values displayed in the heatmaps represent Spearman correlation coefficients (*ρ*), with positive and negative values indicating positive and inverse correlations, respectively. Asterisks indicate statistical significance (*P < 0.05, **P < 0.01, ***P < 0.001).

Spearman correlation analyses were performed to explore the clinical correlates of MONO-derived dynamic indicators (Figure 8).

Both delta MONO and slope MONO showed weak-to-moderate correlations with parameters reflecting systemic inflammation, coagulation dysfunction, renal impairment, and liver functional status. Higher MONO dynamic indices were positively associated with WBC, creatinine, and coagulation abnormalities, while showing negative correlations with albumin and PTA.

Furthermore, changes in MONO dynamics were consistently associated with parallel alterations in inflammatory and coagulation parameters during follow-up, supporting the potential relationship between early MONO elevation and systemic disease progression in ALF.

### 3.13 Sensitivity analysis

To assess the robustness of the primary findings, sensitivity analysis was first performed by re-including one patient who had been excluded from the main cohort because of pre-existing chemotherapy-related bone marrow suppression and long-term abnormal blood cell counts. After inclusion, the cohort consisted of 47 patients. The overall MONO trajectory pattern remained consistent, with a significant group-by-time interaction observed in all linear mixed-effects models (Model 1: *P* = 0.003; Model 2: *P* = 0.003; Model 3: *P* < 0.001) (Supplementary Figure S8). Consistently, delta MONO remained significantly associated with 28-day mortality in most adjusted logistic regression models, whereas slope MONO continued to show a positive but non-significant association (Supplementary Tables S9–S10), indicating that the main findings were not materially affected by this exclusion.

To further assess whether the observed association was primarily driven by the greater burden of sepsis among non-survivors, an additional sensitivity analysis was conducted after excluding patients who developed sepsis. The longitudinal MONO trajectory remained different between survivors and non-survivors in the non-sepsis subgroup (Supplementary Figure S9). In addition, delta MONO remained significantly associated with 28-day mortality across the evaluated multivariable models, with effect estimates broadly consistent with those observed in the primary analysis (Supplementary Table S11). In contrast, the associations of baseline MONO and slope MONO were less consistent across models. These findings suggest that the association between delta MONO and mortality was not solely attributable to the higher prevalence of sepsis among non-survivors.

Given the marked age difference between survivors and non-survivors, age-related sensitivity analyses were also performed. Patients were stratified into younger and older groups according to the median age of the cohort, and longitudinal MONO trajectories were reassessed separately within each age stratum. The group-by-time interaction was more evident in the older subgroup, whereas the corresponding estimates in the younger subgroup were directionally similar but did not consistently reach statistical significance (Supplementary Figure S10 and Supplementary Table S12). Consistent with these findings, age-stratified Firth logistic regression showed that delta MONO was significantly associated with 28-day mortality in the older subgroup but not in the younger subgroup (Supplementary Table S13). However, a formal interaction analysis using age as a continuous variable showed no significant interaction between age and delta MONO (OR = 1.005, 95% CI: 0.991–1.019, *P* = 0.470) (Supplementary Table S14), providing no statistical evidence that age significantly modified the association between delta MONO and 28-day mortality.

## 4 Discussion

In this exploratory longitudinal study of rigorously defined ALF, we demonstrated that early changes in peripheral MONO counts were associated with 28-day mortality, whereas baseline MONO levels alone did not significantly distinguish survivors from non-survivors. Evidence for an association between MONO dynamics and outcome was observed across several complementary analytical approaches, although the strength and consistency of the associations differed among individual MONO-derived measures. Among the evaluated MONO-derived parameters, delta MONO showed the most consistent association with mortality, suggesting that early changes in circulating MONO may provide clinically relevant information beyond a single baseline measurement. Exploratory prognostic analyses further indicated that incorporating MONO-derived dynamic indicators may provide complementary information to conventional liver failure scores, although these findings require validation in larger independent cohorts.

ALF is a rapidly progressive and potentially fatal syndrome characterized by acute loss of hepatic function, coagulopathy, hepatic encephalopathy, and frequent development of systemic complications in patients without pre-existing cirrhosis (17, 18). Despite advances in intensive care management, transplantation strategies, and etiology-specific interventions, short-term mortality remains substantial, particularly among patients complicated by infection, renal dysfunction, and multiorgan failure (19, 20). Current prognostic systems, including the KCC and MELD-based scores, remain important tools for clinical decision-making; however, these approaches mainly rely on static clinical and biochemical measurements obtained at specific time points and may not fully capture the rapidly evolving biological processes underlying ALF progression (17, 21). Therefore, dynamic biomarkers reflecting ongoing disease evolution may provide additional prognostic information.

An important consideration in retrospective ALF studies is the accurate definition of the study population. In routine clinical practice, patients labeled as “acute liver failure” may include heterogeneous conditions such as acute-on-chronic liver failure, subacute liver failure, hypoperfusion-related liver injury, sepsis-associated hepatic dysfunction, malignancy-related liver abnormalities, or isolated coagulation disorders. These conditions differ substantially in pathophysiological mechanisms, inflammatory responses, and clinical management strategies (20). Recent phenotyping studies have further emphasized the biological heterogeneity hidden within conventionally defined ALF populations, demonstrating distinct clinical trajectories among patients initially classified under the same diagnostic category (22).

In the present study, we addressed this challenge by first identifying ICD-coded ALF cases from a real-world hospital population and subsequently performing detailed chart review according to predefined diagnostic criteria. This process substantially reduced diagnostic heterogeneity and resulted in a rigorously defined ALF cohort. Although strict case verification inevitably reduced the final sample size, it improved the biological interpretability of subsequent analyses by minimizing the inclusion of patients with secondary liver injury or non-ALF conditions.

ALF is increasingly recognized not only as a consequence of hepatocellular destruction but also as a systemic immune disorder involving complex interactions between inflammatory activation, immune regulation, and organ dysfunction (23–26). Among circulating immune cells, MONO and macrophage-lineage cells play important roles in coordinating inflammatory responses, antigen presentation, tissue injury, and repair processes (26). Previous studies have demonstrated that alterations in MONO functional states, including reduced human leukocyte antigen-DR expression, are associated with disease severity and outcomes in ALF (24). These findings suggest that MONO-related immune alterations may reflect clinically meaningful changes in host response during ALF progression.

However, previous investigations have primarily focused on MONO functional characteristics or immune phenotypes measured at specific time points. In contrast, our study evaluated routinely available peripheral MONO counts from serial clinical measurements and focused on their temporal evolution during the early phase of ALF. Importantly, baseline MONO values were comparable between survivors and non-survivors, whereas early increases in MONO during follow-up were associated with mortality. This finding supports the concept that immune-cell dynamics may provide information that cannot be captured by single baseline measurements.

The biological interpretation of increasing circulating MONO during ALF progression requires careful consideration. Rising MONO levels may reflect multiple overlapping processes, including enhanced recruitment of circulating myeloid cells, systemic inflammatory activation, infection-related immune responses, or altered immune regulation during severe illness. Previous studies have demonstrated that monocyte and macrophage responses in ALF are highly dynamic and may shift between inflammatory and reparative states depending on disease stage (27–29). Importantly, systemic inflammatory activation in ALF may coexist with compensatory immune suppression rather than representing sustained immune activation alone. In this setting, circulating monocytes may undergo functional deactivation, including reduced HLA-DR expression, impaired antigen presentation, and attenuated responsiveness to subsequent inflammatory stimulation (30). Thus, an increase in circulating MONO does not necessarily indicate preserved or enhanced monocyte function; numerical expansion or mobilization may coexist with qualitative immune dysfunction. Therefore, increasing peripheral MONO should not be interpreted as a direct marker of pathogenic activity alone, but rather as a potential indicator of evolving systemic immune status.

The association between MONO dynamics and mortality may also be partly explained by the greater burden of infection and organ dysfunction observed among non-survivors. In our cohort, patients with poor outcomes more frequently experienced sepsis, respiratory infection, renal failure, and respiratory failure, and required more intensive organ support. ALF shares several features with other critical illnesses, particularly sepsis, where dysregulated innate immune responses, persistent inflammation, and immune dysfunction contribute to disease progression (17, 21, 23). Although ALF and sepsis represent distinct clinical entities, they exhibit overlapping characteristics of systemic inflammation, immune dysregulation, and multiorgan failure. Experimental and clinical evidence in acetaminophen-induced ALF further suggests that circulating monocytes can acquire an anti-inflammatory, hyporesponsive phenotype, with attenuated NF-*κ*B signaling and reduced TNF-α and IL-6 responses to LPS stimulation despite ongoing systemic inflammation (31). This functional reprogramming provides a plausible link between rising circulating MONO, immunoparalysis, and increased susceptibility to secondary infection. In this context, increasing peripheral MONO may reflect a broader systemic inflammatory and immune response associated with disease severity and organ dysfunction rather than liver injury alone. Similar observations have been reported in sepsis, where MONO dysfunction, including altered antigen presentation and immune paralysis, has been linked to clinical outcomes (32, 33).

Given the rapidly progressive clinical course of ALF, clinically meaningful immune and organ dysfunction may evolve within the first several days after presentation (1). The additional sensitivity analyses provide further context for interpretation of the observed MONO–outcome association. First, the shorter-window analysis demonstrated that divergence in MONO trajectories was already detectable within the first 72 h after admission, reducing the possibility that the primary 7-day findings were driven predominantly by terminal biological changes occurring close to death. However, because 72 h MONO-derived indicators still require serial post-admission measurements, they should be interpreted as early longitudinal prognostic markers rather than baseline predictors. Second, the association between delta MONO and mortality remained evident after exclusion of patients who developed sepsis, suggesting that the observed relationship was not solely attributable to infection-related monocyte activation or immune dysfunction. Finally, although age-stratified analyses suggested a more apparent association in older patients, the formal age × delta MONO interaction was not statistically significant, providing no evidence that age materially modified the association. Taken together, these analyses support the robustness of the direction of the primary findings while also emphasizing the exploratory nature of subgroup and sensitivity analyses in this small cohort.

The significance of the 72 h MONO-derived longitudinal prognostic indicators should be considered in the context of the temporal evolution of innate immune responses during acute liver injury. Following hepatocellular injury, damage-associated molecular patterns activate innate immune pathways and chemokine signals that promote circulating monocyte recruitment. In experimental acetaminophen-induced acute liver injury, CCR2-positive monocytes begin to infiltrate the liver as early as 8–12 h after injury, with substantial accumulation of monocyte-derived macrophages within 12–24 h (34). Over the subsequent 24–72 h, the hepatic myeloid compartment continues to evolve, with recruited monocytes/macrophages exhibiting temporal changes in inflammatory and repair-associated phenotypes (35). Although this experimental time course cannot be directly mapped onto the post-admission course of patients with etiologically heterogeneous ALF, these observations support the biological plausibility that monocyte recruitment and remodeling are initiated rapidly and evolve over the first several days of acute liver injury.

This temporal context is particularly relevant to the present cohort, in which non-survivors had a median survival of only 4 days. In such rapidly progressive ALF, waiting for a complete 7-day trajectory would have limited clinical relevance for patients who deteriorate early, whereas serial measurements obtained within the first 72 h may provide an earlier window for prognostic reassessment. The finding that outcome-associated MONO divergence was already detectable within this interval suggests that the 72 h indicators may capture an evolving adverse trajectory rather than merely changes occurring during the later, near-death phase. Their potential significance therefore lies not in replacing established admission-based prognostic scores, but in providing an early dynamic signal that may help update risk assessment during the initial course of hospitalization. For patients who survive beyond this early window, continued longitudinal monitoring may remain informative by showing whether peripheral MONO trajectories stabilize or decline, as observed more often in survivors, or continue to rise, as observed more often in non-survivors.

Coagulation dysfunction represents another important component of ALF pathophysiology and is closely interconnected with systemic inflammation. In our cohort, non-survivors showed more severe coagulation abnormalities, including lower PTA, higher INR, and prolonged APTT. Moreover, longitudinal analysis demonstrated different PTA trajectories between survivors and non-survivors. The interaction between coagulation disturbance and immune activation has been increasingly recognized in severe liver injury, as inflammatory responses, endothelial dysfunction, and coagulation abnormalities may mutually reinforce each other during disease progression (36, 37). In this context, the observation that MONO-derived dynamic indicators provided complementary prognostic information when considered together with PTA is biologically plausible. However, our findings should not be interpreted as evidence that MONO dynamics outperform established coagulation-based prognostic systems, but rather suggest that immune-related information may capture additional aspects of disease evolution not fully reflected by conventional liver function parameters.

Beyond routine blood-cell measurements, emerging evidence has highlighted the importance of MONO–macrophage biology in ALF. Circulating macrophage activation markers, including soluble CD163 and other immune-related indicators, have been associated with disease severity and mortality in liver failure (38, 39). Compared with these mechanistically specific markers, peripheral MONO counts are less specific but have substantial practical advantages because they are routinely available, inexpensive, and repeatedly measured in standard clinical care. Therefore, although MONO count alone is unlikely to serve as a definitive immune biomarker, longitudinal MONO patterns may represent a clinically accessible approach for capturing dynamic immune alterations during ALF.

The potential value of temporal immune monitoring is consistent with previous studies in critical illness demonstrating that longitudinal hematological trajectories may provide stronger prognostic information than isolated measurements (40). A single laboratory value represents only one snapshot of the host response, whereas serial changes may integrate cumulative biological processes occurring during disease progression. In ALF, where clinical deterioration often occurs rapidly within days after admission, monitoring early immune trajectories may provide a window for identifying patients at higher risk of adverse outcomes and may complement existing clinical decision-making frameworks.

From a clinical perspective, the findings of this study have several potential implications. First, routine blood counts are universally available in hospitalized patients, making MONO dynamics a potentially feasible marker for repeated risk assessment without requiring specialized immune profiling techniques. Second, the identification of patients with progressive MONO elevation may help recognize individuals with evolving systemic inflammatory or immune dysregulation patterns who may benefit from closer monitoring and early escalation of supportive management. Third, combining immune trajectory information with conventional indicators such as coagulation parameters may provide a more comprehensive assessment of ALF progression. Nevertheless, these implications remain exploratory, and prospective validation is required before MONO dynamics can be incorporated into routine clinical practice.

This study has several strengths. First, the cohort was derived from a broad ICD-coded population and subsequently refined through detailed clinical review, resulting in a rigorously defined ALF cohort rather than a heterogeneous mixture of ALF, acute-on-chronic liver failure, secondary hepatic injury, and isolated coagulation abnormalities. This approach improved the biological interpretability of the observed associations. Second, the study evaluated longitudinal immune changes during the first week after admission and additionally confirmed the main trajectory pattern within a shorter 72 h window, providing complementary assessment of the temporal robustness of the observed association. Third, multiple complementary analytical approaches were applied, including longitudinal trajectory analysis, linear mixed-effects modeling, patient-level dynamic indicators, trajectory-based clustering, regression analysis, exploratory prognostic modeling, and sensitivity analysis. The consistency of findings across these approaches provides additional support for the observed association between MONO dynamics and short-term mortality. Fourth, because peripheral MONO counts are obtained from routine blood tests, MONO-derived dynamic indicators may have potential translational value if validated in larger cohorts.

Several limitations should be acknowledged. First, the sample size was relatively small, reflecting the rarity of rigorously defined ALF. In particular, the limited number of outcome events constrained the precision and complexity of multivariable modeling and may have increased the risk of overfitting. Therefore, the regression and prognostic modeling results should be interpreted as exploratory, and external validation in larger multicenter cohorts is required. Second, this retrospective single-center study may be subject to inherent selection bias, and the generalizability of our findings needs further confirmation. Third, peripheral MONO counts are indirect indicators and do not capture monocyte functional states or immune phenotypes. In addition, because non-survivors experienced early deaths, longitudinal measurements obtained over the original 7-day window may in some patients have captured biological changes occurring relatively close to death. Although the additional 72 h analysis demonstrated that outcome-associated MONO divergence was already detectable within the first three days after admission, these dynamic measures still require serial post-admission observations and should therefore be interpreted as early prognostic markers rather than baseline predictors. Finally, although significant associations were observed between MONO dynamics and outcomes, the observational design prevents causal inference, and further mechanistic studies are needed to clarify the biological basis of these findings.

## 5 Conclusion

In this exploratory longitudinal study of rigorously defined ALF, increases in peripheral MONO during the first week after admission were consistently associated with 28-day mortality, whereas baseline MONO alone showed limited prognostic value. Among the evaluated MONO-derived measures, delta MONO showed the most consistent association with mortality, while the findings for slope MONO were less stable across multivariable models. Notably, outcome-associated MONO trajectory divergence was already detectable within the first 72 h after admission. These findings suggest that temporal MONO changes may provide additional information beyond single baseline measurements and conventional clinical assessments. Dynamic monitoring of peripheral MONO, an easily accessible parameter from routine blood testing, may represent a simple adjunctive approach for early longitudinal prognostic assessment in ALF. However, these findings remain exploratory and require validation in larger multicenter cohorts with standardized longitudinal sampling and integrated immune profiling.

## Data Availability

The raw data supporting the conclusions of this article will be made available by the authors, without undue reservation.
