# Supplementary material for: Early peripheral monocyte dynamics are associated with 28-day mortality in acute liver failure: an exploratory longitudinal cohort study
Source: Front Med (Lausanne). 2026 Sep 10;13:1945730. doi: 10.3389/fmed.2026.1945730 (PMC13600835; doi:10.3389/fmed.2026.1945730)
Supplement: Supplementary file 1 [file Table1.docx]

**Table S1 Baseline characteristics according to exploratory MONO trajectory-informed clusters**

| **Variable** | **Total**  **(n=46)** | **Class 1**  **(n=8)** | **Class 2**  **(n=38)** | **P value** |
| --- | --- | --- | --- | --- |
| 28-day mortality, n (%) | 23 (50.0%) | 7 (87.5%) | 16 (42.1%) | **0.047** |
| Male sex, n (%) | 20 (43.5%) | 5 (62.5%) | 15 (39.5%) | 0.267 |
| Age | 48.41 ± 18.04 | 51.75 ± 18.08 | 47.71 ± 18.19 | 0.579 |
| Ascites, n (%) | 25 (54.3%) | 5 (62.5%) | 20 (52.6%) | 0.710 |
| Pleural effusion, n (%) | 11 (23.9%) | 1 (12.5%) | 10 (26.3%) | 0.658 |
| Sepsis, n (%) | 12 (26.1%) | 4 (50.0%) | 8 (21.1%) | 0.178 |
| Electrolyte disturbance, n (%) | 33 (71.7%) | 6 (75.0%) | 27 (71.1%) | 1.000 |
| Gastrointestinal bleeding, n (%) | 9 (19.6%) | 2 (25.0%) | 7 (18.4%) | 0.645 |
| Respiratory tract infection, n (%) | 14 (30.4%) | 5 (62.5%) | 9 (23.7%) | **0.044** |
| Abdominal infection, n (%) | 7 (15.2%) | 3 (37.5%) | 4 (10.5%) | 0.089 |
| Renal failure, n (%) | 17 (37.0%) | 5 (62.5%) | 12 (31.6%) | 0.125 |
| Respiratory failure, n (%) | 18 (39.1%) | 8 (100.0%) | 10 (26.3%) | **<0.001** |
| ICU admission, n (%) | 11 (23.9%) | 3 (37.5%) | 8 (21.1%) | 0.374 |
| Mechanical ventilation, n (%) | 13 (28.3%) | 4 (50.0%) | 9 (23.7%) | 0.196 |
| Vasopressor use, n (%) | 12 (26.1%) | 2 (25.0%) | 10 (26.3%) | 1.000 |
| CRRT, n (%) | 9 (19.6%) | 3 (37.5%) | 6 (15.8%) | 0.176 |
| Artificial liver support therapy, n (%) | 28 (60.9%) | 6 (75.0%) | 22 (57.9%) | 0.453 |
| WBC | 7.90 (5.78, 12.18) | 14.70 (11.45, 19.22) | 6.80 (5.53, 10.12) | **0.002** |
| RBC | 4.41 ± 0.82 | 3.98 ± 0.97 | 4.51 ± 0.77 | 0.180 |
| HGB | 133.14 ± 21.95 | 122.75 ± 26.36 | 135.33 ± 20.65 | 0.236 |
| HCT | 38.93 ± 7.01 | 35.60 ± 7.65 | 39.63 ± 6.76 | 0.199 |
| MCV | 89.81 ± 8.42 | 90.25 ± 8.88 | 89.72 ± 8.44 | 0.880 |
| MCH | 30.37 ± 3.35 | 29.75 ± 4.56 | 30.50 ± 3.10 | 0.668 |
| MCHC | 339.50 (329.25, 347.00) | 347.00 (341.50, 347.50) | 335.50 (328.25, 345.00) | 0.065 |
| RDW-CV | 15.35 (13.83, 17.70) | 16.60 (14.53, 19.60) | 15.15 (13.72, 17.70) | 0.172 |
| RDW-SD | 47.40 (41.77, 54.00) | 49.05 (38.15, 54.00) | 47.05 (41.77, 55.20) | 0.851 |
| PLT | 135.50 (90.75, 197.50) | 92.50 (71.75, 158.25) | 139.50 (107.25, 197.50) | 0.218 |
| PDW | 15.05 ± 2.70 | 15.99 ± 3.07 | 14.86 ± 2.62 | 0.357 |
| PCT-PLT | 0.15 (0.11, 0.20) | 0.11 (0.07, 0.15) | 0.17 (0.12, 0.21) | 0.095 |
| MPV | 11.11 ± 1.26 | 11.18 ± 1.64 | 11.09 ± 1.19 | 0.899 |
| LYMPH | 1.15 (0.71, 1.66) | 1.24 (0.61, 1.99) | 1.15 (0.80, 1.58) | 0.942 |
| MONO | 0.80 ± 0.44 | 1.55 ± 0.45 | 0.64 ± 0.23 | **<0.001** |
| NEUT | 5.39 (3.76, 9.55) | 11.57 (7.29, 16.90) | 5.12 (3.23, 7.71) | **0.005** |
| EO | 0.10 (0.02, 0.14) | 0.04 (0.02, 0.06) | 0.10 (0.02, 0.17) | 0.100 |
| PT | 29.10 (22.47, 46.38) | 37.60 (26.30, 72.30) | 29.10 (22.25, 39.25) | 0.284 |
| PTA | 27.11 ± 12.35 | 25.45 ± 16.41 | 27.46 ± 11.57 | 0.750 |
| INR | 2.61 (1.96, 3.46) | 2.52 (1.98, 5.48) | 2.61 (1.96, 3.30) | 0.685 |
| APTT | 45.55 (37.17, 55.35) | 58.90 (52.62, 67.80) | 42.45 (35.83, 53.02) | **0.008** |
| TT | 20.55 (16.15, 24.25) | 23.85 (16.90, 24.88) | 20.40 (16.15, 23.43) | 0.478 |
| AT-III | 29.25 (17.23, 61.05) | 25.70 (20.02, 67.00) | 32.84 (17.23, 57.90) | 0.977 |
| FDP | 13.07 (2.24, 32.39) | 39.80 (5.90, 83.83) | 11.40 (1.86, 29.21) | 0.182 |
| D-D | 227.33 (5.85, 1670.75) | 16.42 (6.27, 177.40) | 317.00 (3.74, 1955.50) | 0.271 |
| FIB-C | 1.31 (0.94, 1.93) | 0.96 (0.67, 1.31) | 1.39 (1.05, 2.02) | **0.036** |
| ALT | 1140.00 (583.50, 2369.25) | 963.20 (424.00, 2717.50) | 1245.17 (599.25, 2171.00) | 0.618 |
| AST | 1025.50 (365.25, 2125.50) | 1279.80 (129.75, 1952.93) | 1002.23 (415.75, 2125.50) | 0.680 |
| TP | 59.00 ± 9.66 | 56.24 ± 15.38 | 59.58 ± 8.17 | 0.568 |
| ALB | 33.78 ± 5.85 | 34.20 ± 8.68 | 33.69 ± 5.23 | 0.876 |
| GLB | 25.43 ± 6.50 | 21.54 ± 9.88 | 26.25 ± 5.38 | 0.227 |
| TBIL | 235.01 (122.92, 414.50) | 188.65 (150.38, 476.60) | 253.20 (113.52, 414.50) | 0.701 |
| DBIL | 130.85 (67.50, 285.80) | 124.30 (76.70, 294.35) | 136.55 (58.20, 268.03) | 1.000 |
| IBIL | 94.55 (35.90, 192.90) | 90.80 (67.78, 188.32) | 98.33 (35.90, 192.90) | 0.908 |
| CHE | 3958.50 (3067.50, 6132.50) | 5632.00 (3824.25, 6880.00) | 3770.00 (3050.00, 5505.00) | 0.317 |
| ALP | 149.50 (115.25, 188.50) | 133.50 (116.00, 147.25) | 158.53 (115.50, 197.50) | 0.240 |
| GGT | 115.31 (52.25, 161.00) | 60.00 (48.25, 132.25) | 121.22 (59.25, 166.50) | 0.258 |
| GLU | 5.92 (3.90, 8.01) | 4.63 (2.79, 12.79) | 5.95 (4.02, 7.88) | 0.572 |
| TG | 2.82 (2.10, 4.08) | 3.92 (0.47, 4.26) | 2.75 (2.13, 4.08) | 0.942 |
| TC | 1.31 (0.76, 2.30) | 2.27 (2.01, 2.71) | 1.12 (0.62, 2.18) | 0.018 |
| HDL-C | 0.62 (0.43, 0.90) | 1.07 (0.66, 1.34) | 0.61 (0.43, 0.76) | 0.077 |
| LDL-C | 1.63 (1.08, 2.43) | 1.93 (1.00, 2.62) | 1.63 (1.14, 2.43) | 0.674 |
| K | 3.85 (3.47, 4.33) | 3.91 (3.43, 4.58) | 3.85 (3.47, 4.28) | 0.817 |
| Na | 137.72 ± 4.92 | 136.00 ± 5.45 | 138.08 ± 4.81 | 0.342 |
| Cl | 101.21 ± 7.00 | 96.34 ± 8.28 | 102.23 ± 6.35 | 0.091 |
| BUN | 4.10 (2.63, 8.42) | 6.34 (3.47, 13.34) | 3.92 (2.63, 7.71) | 0.393 |
| CRE | 66.00 (47.25, 96.68) | 108.90 (47.00, 141.00) | 63.50 (48.25, 92.50) | 0.317 |
| PCT | 1.00 (0.53, 2.98) | 1.36 (0.54, 3.47) | 0.94 (0.53, 1.99) | 0.653 |
| NLR | 5.57 (2.43, 10.89) | 13.50 (5.51, 26.57) | 4.85 (2.34, 9.57) | 0.074 |
| MLR | 0.58 (0.35, 1.07) | 1.51 (0.75, 2.38) | 0.51 (0.32, 0.86) | **0.011** |
| PLR | 119.16 (76.92, 201.98) | 108.21 (95.36, 146.67) | 130.02 (76.92, 201.98) | 0.618 |
| SII | 748.27 (390.50, 1432.90) | 1003.41 (573.56, 1882.57) | 738.38 (380.49, 1325.57) | 0.191 |
| SIRI | 3.97 (1.29, 7.89) | 19.57 (7.30, 45.59) | 3.03 (1.17, 6.40) | **0.001** |
