# Supplementary material for: Early peripheral monocyte dynamics are associated with 28-day mortality in acute liver failure: an exploratory longitudinal cohort study
Source: Front Med (Lausanne). 2026 Sep 10;13:1945730. doi: 10.3389/fmed.2026.1945730 (PMC13600835; doi:10.3389/fmed.2026.1945730)
Supplement: Supplementary file 2 [file Table10.docx]

**Table S10 Sensitivity analysis of multivariable logistic regression models for MONO-derived indicators after re-including the excluded patient**

| **Exposure** | **Model 1** | **Model 2** | **Model 3A** | **Model 3B** | **Model 3C** | **Model 3D** | **Model 3E** | **Model 3F** |
| --- | --- | --- | --- | --- | --- | --- | --- | --- |
| MONO | 1.099  (0.960, 1.295), P = 0.205 | 1.232  (1.011, 1.620), P = 0.079 | 1.174  (0.943, 1.605), P = 0.225 | 1.316  (1.025, 1.841), P = 0.068 | 1.242  (0.920, 1.809), P = 0.202 | 1.347  (1.010, 1.990), P = 0.090 | 1.669  (1.178, 2.718),  **P = 0.013** | 1.318  (0.984, 1.914), P = 0.095 |
| Delta MONO | 1.202  (1.052, 1.434), **P = 0.018** | 1.268  (1.071, 1.612),  **P = 0.018** | 1.282  (1.057, 1.709), **P = 0.035** | 1.239  (1.032, 1.617), P = 0.052 | 1.263  (1.034, 1.726), P = 0.063 | 1.355  (1.094, 1.929), **P = 0.026** | 1.321  (1.077, 1.839),  **P = 0.031** | 1.351  (1.078, 1.964), **P = 0.033** |
| Slope MONO | 1.041  (0.997, 1.104), P = 0.113 | 1.047  (0.999, 1.113),  P = 0.082 | 1.046  (0.989, 1.129), P = 0.163 | 1.031  (0.979, 1.103), P = 0.293 | 1.035  (0.983, 1.112), P = 0.226 | 1.054  (0.997, 1.140), P = 0.099 | 1.043  (0.990, 1.118), P = 0.140 | 1.048  (0.992, 1.129), P = 0.107 |

Model 1: Raw model

Model 2: Age + Gender

Model 3A: Age + Gender + WBC + MELD

Model 3B: Age + Gender + NLR + MELD

Model 3C: Age + Gender + Sepsis + MELD

Model 3D: Age + Gender + WBC + PTA

Model 3E: Age + Gender + NLR + PTA

Model 3F: Age + Gender + Sepsis + PTA
