# Supplementary material for: Early peripheral monocyte dynamics are associated with 28-day mortality in acute liver failure: an exploratory longitudinal cohort study
Source: Front Med (Lausanne). 2026 Sep 10;13:1945730. doi: 10.3389/fmed.2026.1945730 (PMC13600835; doi:10.3389/fmed.2026.1945730)
Supplement: Supplementary file 3 [file Table11.docx]

**Table S11 Sensitivity analysis of multivariable logistic regression models for MONO-derived indicators in the non-sepsis subgroup**

| **Exposure** | **Model 1** | **Model 2** | **Model 3A** | **Model 3B** | **Model 3C** | **Model 3D** |
| --- | --- | --- | --- | --- | --- | --- |
| MONO | 1.085  (0.957, 1.269),  P = 0.212 | 1.176  (0.989, 1.513),  P = 0.070 | 1.115  (0.934, 1.469),  P = 0.244 | 1.217  (0.985, 1.649),  P = 0.075 | 1.245  (0.975, 1.783),  P = 0.090 | 1.497  (1.096, 2.255),  **P = 0.007** |
| Delta MONO | 1.178  (1.039, 1.395),  **P = 0.007** | 1.212  (1.045, 1.494),  **P = 0.009** | 1.208  (1.032, 1.537),  **P = 0.016** | 1.174  (1.006, 1.474),  **P = 0.040** | 1.254  (1.058, 1.662),  **P = 0.007** | 1.228  (1.041, 1.607),  **P = 0.012** |
| Slope MONO | 1.034  (0.994, 1.093),  P = 0.099 | 1.037  (0.996, 1.094),  P = 0.080 | 1.034  (0.989, 1.105),  P = 0.145 | 1.022  (0.978, 1.083),  P = 0.342 | 1.041  (0.996, 1.113),  P = 0.075 | 1.033  (0.989, 1.095),  P = 0.145 |

Model 1: Raw model

Model 2: Age + Gender

Model 3A: Age + Gender + WBC + MELD

Model 3B: Age + Gender + NLR + MELD

Model 3C: Age + Gender + WBC + PTA

Model 3D: Age + Gender + NLR + PTA
