# Supplementary material for: Early peripheral monocyte dynamics are associated with 28-day mortality in acute liver failure: an exploratory longitudinal cohort study
Source: Front Med (Lausanne). 2026 Sep 10;13:1945730. doi: 10.3389/fmed.2026.1945730 (PMC13600835; doi:10.3389/fmed.2026.1945730)
Supplement: Supplementary file 4 [file Table12.docx]

**Table S12 Age-stratified sensitivity analysis of longitudinal monocyte trajectories using linear mixed-effects models**

|  |  | Model 1 (95% CI) | P value | Model 2 (95% CI) | P value | Model 3 (95% CI) | P value |
| --- | --- | --- | --- | --- | --- | --- | --- |
| Younger group | Intercept | 0.679 (0.485, 0.874) | **<0.001** | 0.632 (0.406, 0.857) | **<0.001** | 0.296 (0.042, 0.550) | **0.023** |
|  | Day | -0.001 (-0.003, 0.001) | 0.463 | -0.001 (-0.003, 0.001) | 0.490 | -0.002 (-0.004, 0.000) | 0.069 |
|  | Outcome | 0.424 (0.009, 0.839) | **0.045** | 0.445 (0.031, 0.859) | **0.036** | 0.191 (-0.181, 0.563) | 0.307 |
|  | Day × Outcome | 0.006 (-0.002, 0.013) | 0.122 | 0.006 (-0.002, 0.013) | 0.123 | 0.007 (-0.000, 0.013) | 0.055 |
| Older group | Intercept | 0.721 (0.281, 1.161) | **0.002** | 0.613 (0.094, 1.131) | **0.022** | 0.190 (-0.314, 0.694) | 0.449 |
|  | Day | -0.001 (-0.006, 0.003) | 0.567 | -0.001 (-0.006, 0.004) | 0.633 | -0.002 (-0.006, 0.002) | 0.381 |
|  | Outcome | 0.015 (-0.489, 0.519) | 0.953 | 0.042 (-0.461, 0.546) | 0.865 | -0.156 (-0.626, 0.314) | 0.504 |
|  | Day × Outcome | 0.006 (0.000, 0.012) | **0.045** | 0.006 (-0.000, 0.012) | 0.052 | 0.006 (0.001, 0.011) | **0.015** |

Model 1: Unadjusted model
Model 2: Adjusted for gender
Model 3: Adjusted for gender and WBC
