# Supplementary material for: Early peripheral monocyte dynamics are associated with 28-day mortality in acute liver failure: an exploratory longitudinal cohort study
Source: Front Med (Lausanne). 2026 Sep 10;13:1945730. doi: 10.3389/fmed.2026.1945730 (PMC13600835; doi:10.3389/fmed.2026.1945730)
Supplement: Supplementary file 5 [file Table13.docx]

**Table S13 Age-stratified Firth logistic regression analyses of MONO-derived indicators associated with 28-day mortality**

|  | **Younger group** | | **Older group** | |
| --- | --- | --- | --- | --- |
| **Exposure** | **Model 1** | **Model 2** | **Model 1** | **Model 2** |
| MONO | 1.344  (1.048, 1.880),  **P = 0.018** | 1.407  (1.066, 2.631),  **P = 0.013** | 0.988  (0.846, 1.215),  P = 0.884 | 0.996  (0.853, 1.219),  P = 0.963 |
| Delta MONO | 1.098  (0.928, 1.346),  P = 0.266 | 1.112  (0.932, 1.374),  P = 0.235 | 1.356  (1.048, 1.993),  **P = 0.014** | 1.337  (1.047, 1.946),  **P = 0.013** |
| Slope MONO | 1.013  (0.958, 1.061),  P = 0.576 | 1.016  (0.959, 1.070),  P = 0.515 | 1.081  (1.005, 1.215),  **P = 0.035** | 1.080  (1.009, 1.197),  **P = 0.025** |

Model 1: Raw model

Model 2: Adjusted for gender
