# Supplementary material for: Early peripheral monocyte dynamics are associated with 28-day mortality in acute liver failure: an exploratory longitudinal cohort study
Source: Front Med (Lausanne). 2026 Sep 10;13:1945730. doi: 10.3389/fmed.2026.1945730 (PMC13600835; doi:10.3389/fmed.2026.1945730)
Supplement: Supplementary file 6 [file Table14.docx]

**Table S14 Interaction analysis between age and delta MONO for the association with 28-day mortality in patients with acute liver failure**

|  | **OR (95% CI)** | **P value** |
| --- | --- | --- |
| Delta MONO | 0.950 (0.513, 1.971) | 0.871 |
| Age | 1.091 (1.037, 1.174) | **<0.001** |
| Delta MONO × Age interaction | 1.005 (0.991, 1.019) | 0.470 |
