# Supplementary material for: Early peripheral monocyte dynamics are associated with 28-day mortality in acute liver failure: an exploratory longitudinal cohort study
Source: Front Med (Lausanne). 2026 Sep 10;13:1945730. doi: 10.3389/fmed.2026.1945730 (PMC13600835; doi:10.3389/fmed.2026.1945730)
Supplement: Supplementary file 7 [file Table2.docx]

**Table S2 Full multivariable logistic regression results for MONO-derived metrics associated with 28-day mortality in acute liver failure**

| **Exposure** | **Model** | **Adjustment** | **Variable** | **OR (95% CI)** | **P value** |
| --- | --- | --- | --- | --- | --- |
| MONO | Model1 | Unadjusted | MONO | 1.120 (0.973, 1.334) | 0.148 |
| MONO | Model2 | Age + Gender | MONO | 1.260 (1.024, 1.682) | 0.065 |
| MONO | Model2 | Age + Gender | Age | 1.122 (1.057, 1.223) | **0.002** |
| MONO | Model2 | Age + Gender | Gender: 1 | 0.174 (0.020, 1.014) | 0.075 |
| MONO | Model3A | Age + Gender + WBC + MELD | MONO | 1.198 (0.955, 1.669) | 0.194 |
| MONO | Model3A | Age + Gender + WBC + MELD | Age | 1.105 (1.041, 1.201) | **0.005** |
| MONO | Model3A | Age + Gender + WBC + MELD | Gender: 1 | 0.155 (0.016, 0.986) | 0.070 |
| MONO | Model3A | Age + Gender + WBC + MELD | WBC | 1.076 (0.865, 1.387) | 0.535 |
| MONO | Model3A | Age + Gender + WBC + MELD | MELD | 1.097 (0.974, 1.243) | 0.126 |
| MONO | Model3B | Age + Gender + NLR + MELD | MONO | 1.370 (1.047, 1.982) | 0.053 |
| MONO | Model3B | Age + Gender + NLR + MELD | Age | 1.138 (1.064, 1.254) | **0.001** |
| MONO | Model3B | Age + Gender + NLR + MELD | Gender: 1 | 0.210 (0.021, 1.444) | 0.137 |
| MONO | Model3B | Age + Gender + NLR + MELD | NLR | 0.972 (0.913, 0.998) | 0.094 |
| MONO | Model3B | Age + Gender + NLR + MELD | MELD | 1.125 (0.994, 1.295) | 0.069 |
| MONO | Model3C | Age + Gender + Sepsis + MELD | MONO | 1.242 (0.920, 1.809) | 0.202 |
| MONO | Model3C | Age + Gender + Sepsis + MELD | Age | 1.095 (1.030, 1.196) | 0.014 |
| MONO | Model3C | Age + Gender + Sepsis + MELD | Gender: 1 | 0.176 (0.014, 1.285) | 0.123 |
| MONO | Model3C | Age + Gender + Sepsis + MELD | Sepsis | 18.459 (0.857, 1972.016) | 0.135 |
| MONO | Model3C | Age + Gender + Sepsis + MELD | MELD | 1.092 (0.946, 1.272) | 0.224 |
| MONO | Model3D | Age + Gender + WBC + PTA | MONO | 1.356 (1.017, 2.006) | 0.084 |
| MONO | Model3D | Age + Gender + WBC + PTA | Age | 1.124 (1.051, 1.237) | **0.004** |
| MONO | Model3D | Age + Gender + WBC + PTA | Gender: 1 | 0.130 (0.012, 0.866) | 0.055 |
| MONO | Model3D | Age + Gender + WBC + PTA | WBC | 1.019 (0.797, 1.327) | 0.883 |
| MONO | Model3D | Age + Gender + WBC + PTA | PTA | 0.928 (0.842, 1.011) | 0.101 |
| MONO | Model3E | Age + Gender + NLR + PTA | MONO | 1.720 (1.203, 2.838) | **0.010** |
| MONO | Model3E | Age + Gender + NLR + PTA | Age | 1.182 (1.083, 1.353) | **0.002** |
| MONO | Model3E | Age + Gender + NLR + PTA | Gender: 1 | 0.109 (0.008, 0.894) | 0.062 |
| MONO | Model3E | Age + Gender + NLR + PTA | NLR | 0.960 (0.903, 0.990) | **0.027** |
| MONO | Model3E | Age + Gender + NLR + PTA | PTA | 0.883 (0.774, 0.971) | **0.024** |
| MONO | Model3F | Age + Gender + Sepsis + PTA | MONO | 1.318 (0.984, 1.914) | 0.095 |
| MONO | Model3F | Age + Gender + Sepsis + PTA | Age | 1.107 (1.037, 1.213) | **0.009** |
| MONO | Model3F | Age + Gender + Sepsis + PTA | Gender: 1 | 0.155 (0.014, 1.069) | 0.085 |
| MONO | Model3F | Age + Gender + Sepsis + PTA | Sepsis | 24.058 (1.343, 2062.594) | 0.076 |
| MONO | Model3F | Age + Gender + Sepsis + PTA | PTA | 0.918 (0.826, 1.000) | 0.067 |
| Delta MONO | Model1 | Unadjusted | Delta MONO | 1.212 (1.058, 1.453) | **0.016** |
| Delta MONO | Model2 | Age + Gender | Delta MONO | 1.273 (1.075, 1.617) | **0.016** |
| Delta MONO | Model2 | Age + Gender | Age | 1.118 (1.052, 1.217) | **0.002** |
| Delta MONO | Model2 | Age + Gender | Gender: 1 | 0.161 (0.015, 1.056) | 0.086 |
| Delta MONO | Model3A | Age + Gender + WBC + MELD | Delta MONO | 1.324 (1.072, 1.851) | **0.035** |
| Delta MONO | Model3A | Age + Gender + WBC + MELD | Age | 1.100 (1.036, 1.197) | **0.007** |
| Delta MONO | Model3A | Age + Gender + WBC + MELD | Gender: 1 | 0.100 (0.005, 0.872) | 0.066 |
| Delta MONO | Model3A | Age + Gender + WBC + MELD | WBC | 1.116 (0.941, 1.375) | 0.232 |
| Delta MONO | Model3A | Age + Gender + WBC + MELD | MELD | 1.098 (0.970, 1.250) | 0.137 |
| Delta MONO | Model3B | Age + Gender + NLR + MELD | Delta MONO | 1.257 (1.042, 1.658) | **0.045** |
| Delta MONO | Model3B | Age + Gender + NLR + MELD | Age | 1.108 (1.046, 1.199) | **0.002** |
| Delta MONO | Model3B | Age + Gender + NLR + MELD | Gender: 1 | 0.215 (0.018, 1.639) | 0.169 |
| Delta MONO | Model3B | Age + Gender + NLR + MELD | NLR | 0.990 (0.941, 1.017) | 0.516 |
| Delta MONO | Model3B | Age + Gender + NLR + MELD | MELD | 1.122 (1.000, 1.277) | 0.056 |
| Delta MONO | Model3C | Age + Gender + Sepsis + MELD | Delta MONO | 1.263 (1.034, 1.726) | 0.063 |
| Delta MONO | Model3C | Age + Gender + Sepsis + MELD | Age | 1.084 (1.022, 1.177) | **0.020** |
| Delta MONO | Model3C | Age + Gender + Sepsis + MELD | Gender: 1 | 0.227 (0.020, 1.601) | 0.169 |
| Delta MONO | Model3C | Age + Gender + Sepsis + MELD | Sepsis | 15.348 (0.690, 1351.471) | 0.147 |
| Delta MONO | Model3C | Age + Gender + Sepsis + MELD | MELD | 1.115 (0.970, 1.308) | 0.137 |
| Delta MONO | Model3D | Age + Gender + WBC + PTA | Delta MONO | 1.373 (1.101, 1.978) | **0.024** |
| Delta MONO | Model3D | Age + Gender + WBC + PTA | Age | 1.108 (1.040, 1.213) | **0.007** |
| Delta MONO | Model3D | Age + Gender + WBC + PTA | Gender: 1 | 0.083 (0.004, 0.800) | 0.063 |
| Delta MONO | Model3D | Age + Gender + WBC + PTA | WBC | 1.089 (0.909, 1.349) | 0.373 |
| Delta MONO | Model3D | Age + Gender + WBC + PTA | PTA | 0.938 (0.855, 1.019) | 0.137 |
| Delta MONO | Model3E | Age + Gender + NLR + PTA | Delta MONO | 1.330 (1.083, 1.852) | **0.028** |
| Delta MONO | Model3E | Age + Gender + NLR + PTA | Age | 1.110 (1.047, 1.206) | **0.003** |
| Delta MONO | Model3E | Age + Gender + NLR + PTA | Gender: 1 | 0.168 (0.011, 1.401) | 0.136 |
| Delta MONO | Model3E | Age + Gender + NLR + PTA | NLR | 0.990 (0.943, 1.017) | 0.493 |
| Delta MONO | Model3E | Age + Gender + NLR + PTA | PTA | 0.919 (0.839, 0.992) | **0.043** |
| Delta MONO | Model3F | Age + Gender + Sepsis + PTA | Delta MONO | 1.351 (1.078, 1.964) | **0.033** |
| Delta MONO | Model3F | Age + Gender + Sepsis + PTA | Age | 1.102 (1.030, 1.211) | **0.016** |
| Delta MONO | Model3F | Age + Gender + Sepsis + PTA | Gender: 1 | 0.138 (0.009, 1.167) | 0.102 |
| Delta MONO | Model3F | Age + Gender + Sepsis + PTA | Sepsis | 34.908 (1.431, 3126.116) | 0.059 |
| Delta MONO | Model3F | Age + Gender + Sepsis + PTA | PTA | 0.901 (0.806, 0.986) | **0.038** |
| Slope MONO | Model1 | Unadjusted | Slope MONO | 1.042 (0.998, 1.107) | 0.105 |
| Slope MONO | Model2 | Age + Gender | Slope MONO | 1.047 (0.999, 1.113) | 0.077 |
| Slope MONO | Model2 | Age + Gender | Age | 1.107 (1.049, 1.194) | **0.002** |
| Slope MONO | Model2 | Age + Gender | Gender: 1 | 0.211 (0.025, 1.174) | 0.105 |
| Slope MONO | Model3A | Age + Gender + WBC + MELD | Slope MONO | 1.049 (0.990, 1.140) | 0.159 |
| Slope MONO | Model3A | Age + Gender + WBC + MELD | Age | 1.091 (1.032, 1.175) | **0.006** |
| Slope MONO | Model3A | Age + Gender + WBC + MELD | Gender: 1 | 0.151 (0.013, 1.003) | 0.078 |
| Slope MONO | Model3A | Age + Gender + WBC + MELD | WBC | 1.125 (0.946, 1.407) | 0.222 |
| Slope MONO | Model3A | Age + Gender + WBC + MELD | MELD | 1.081 (0.962, 1.218) | 0.184 |
| Slope MONO | Model3B | Age + Gender + NLR + MELD | Slope MONO | 1.033 (0.980, 1.105) | 0.268 |
| Slope MONO | Model3B | Age + Gender + NLR + MELD | Age | 1.106 (1.047, 1.190) | **0.002** |
| Slope MONO | Model3B | Age + Gender + NLR + MELD | Gender: 1 | 0.304 (0.035, 1.898) | 0.228 |
| Slope MONO | Model3B | Age + Gender + NLR + MELD | NLR | 0.986 (0.941, 1.012) | 0.349 |
| Slope MONO | Model3B | Age + Gender + NLR + MELD | MELD | 1.119 (1.001, 1.268) | 0.055 |
| Slope MONO | Model3C | Age + Gender + Sepsis + MELD | Slope MONO | 1.035 (0.983, 1.112) | 0.226 |
| Slope MONO | Model3C | Age + Gender + Sepsis + MELD | Age | 1.082 (1.024, 1.166) | **0.014** |
| Slope MONO | Model3C | Age + Gender + Sepsis + MELD | Gender: 1 | 0.266 (0.030, 1.609) | 0.179 |
| Slope MONO | Model3C | Age + Gender + Sepsis + MELD | Sepsis | 16.263 (1.139, 865.107) | 0.087 |
| Slope MONO | Model3C | Age + Gender + Sepsis + MELD | MELD | 1.099 (0.962, 1.273) | 0.170 |
| Slope MONO | Model3D | Age + Gender + WBC + PTA | Slope MONO | 1.056 (0.998, 1.146) | 0.098 |
| Slope MONO | Model3D | Age + Gender + WBC + PTA | Age | 1.094 (1.035, 1.179) | **0.006** |
| Slope MONO | Model3D | Age + Gender + WBC + PTA | Gender: 1 | 0.129 (0.010, 0.894) | 0.065 |
| Slope MONO | Model3D | Age + Gender + WBC + PTA | WBC | 1.114 (0.934, 1.394) | 0.268 |
| Slope MONO | Model3D | Age + Gender + WBC + PTA | PTA | 0.958 (0.885, 1.034) | 0.267 |
| Slope MONO | Model3E | Age + Gender + NLR + PTA | Slope MONO | 1.044 (0.991, 1.119) | 0.132 |
| Slope MONO | Model3E | Age + Gender + NLR + PTA | Age | 1.103 (1.045, 1.185) | **0.002** |
| Slope MONO | Model3E | Age + Gender + NLR + PTA | Gender: 1 | 0.253 (0.026, 1.612) | 0.179 |
| Slope MONO | Model3E | Age + Gender + NLR + PTA | NLR | 0.987 (0.944, 1.013) | 0.367 |
| Slope MONO | Model3E | Age + Gender + NLR + PTA | PTA | 0.934 (0.862, 1.002) | 0.068 |
| Slope MONO | Model3F | Age + Gender + Sepsis + PTA | Slope MONO | 1.048 (0.992, 1.129) | 0.107 |
| Slope MONO | Model3F | Age + Gender + Sepsis + PTA | Age | 1.091 (1.028, 1.183) | **0.013** |
| Slope MONO | Model3F | Age + Gender + Sepsis + PTA | Gender: 1 | 0.193 (0.019, 1.297) | 0.119 |
| Slope MONO | Model3F | Age + Gender + Sepsis + PTA | Sepsis | 31.755 (2.014, 1502.080) | **0.035** |
| Slope MONO | Model3F | Age + Gender + Sepsis + PTA | PTA | 0.922 (0.838, 0.999) | 0.062 |
