# Supplementary material for: Early peripheral monocyte dynamics are associated with 28-day mortality in acute liver failure: an exploratory longitudinal cohort study
Source: Front Med (Lausanne). 2026 Sep 10;13:1945730. doi: 10.3389/fmed.2026.1945730 (PMC13600835; doi:10.3389/fmed.2026.1945730)
Supplement: Supplementary file 8 [file Table3.docx]

**Table S3 Clinical and laboratory characteristics according to 7d delta MONO status**

| **Variable** | **Delta MONO > 0** | **Delta MONO ≤ 0** | **P value** |
| --- | --- | --- | --- |
| 28-day mortality, n (%) | 19 (65.5%) | 4 (23.5%) | **0.006** |
| Gender | 13 (44.8%) | 7 (41.2%) | 0.810 |
| Age | 50.31 ± 19.14 | 45.18 ± 15.99 | 0.335 |
| Ascites | 17 (58.6%) | 8 (47.1%) | 0.447 |
| Pleural effusion | 9 (31.0%) | 2 (11.8%) | 0.172 |
| Sepsis | 10 (34.5%) | 2 (11.8%) | 0.163 |
| Electrolyte disturbance | 21 (72.4%) | 12 (70.6%) | 1.000 |
| Gastrointestinal bleeding | 7 (24.1%) | 2 (11.8%) | 0.450 |
| Respiratory tract infection | 11 (37.9%) | 3 (17.6%) | 0.149 |
| Abdominal infection | 5 (17.2%) | 2 (11.8%) | 1.000 |
| Renal failure | 14 (48.3%) | 3 (17.6%) | **0.038** |
| Respiratory failure | 15 (51.7%) | 3 (17.6%) | **0.022** |
| ICU admission | 11 (37.9%) | 0 (0.0%) | **0.003** |
| Mechanical ventilation | 12 (**41**.4%) | 1 (5.9%) | **0.016** |
| Vasopressor use | 11 (37.9%) | 1 (5.9%) | **0.034** |
| CRRT | 9 (31.0%) | 0 (0.0%) | **0.017** |
| Artificial liver support therapy | 17 (58.6%) | 11 (64.7%) | 0.683 |
| WBC | 8.30 (5.70, 13.10) | 7.20 (6.20, 9.90) | 0.741 |
| RBC | 4.27 ± 0.82 | 4.66 ± 0.79 | 0.118 |
| HGB | 129.08 ± 22.26 | 140.06 ± 20.18 | 0.095 |
| HCT | 37.90 ± 6.86 | 40.68 ± 7.12 | 0.204 |
| MCV | 90.16 ± 8.40 | 89.21 ± 8.67 | 0.719 |
| MCH | 30.03 ± 3.16 | 30.94 ± 3.66 | 0.402 |
| MCHC | 336.00 (329.00, 347.00) | 342.00 (332.00, 350.00) | 0.259 |
| RDW | 15.50 (14.00, 17.70) | 15.10 (13.30, 17.20) | 0.289 |
| SD | 47.30 (42.70, 54.00) | 47.50 (39.60, 51.80) | 0.625 |
| PLT | 140.00 (79.00, 187.00) | 134.00 (108.00, 199.00) | 0.577 |
| PDW | 15.23 ± 2.77 | 14.75 ± 2.65 | 0.565 |
| PLT-PCT | 0.14 (0.08, 0.19) | 0.17 (0.12, 0.21) | 0.412 |
| MPV | 10.97 ± 1.26 | 11.35 ± 1.26 | 0.321 |
| LYMPH | 1.00 (0.68, 1.97) | 1.30 (0.79, 1.43) | 0.311 |
| MONO | 0.67 (0.47, 0.84) | 0.78 (0.66, 0.90) | 0.070 |
| NEUT | 5.46 (3.31, 9.58) | 5.18 (3.90, 8.76) | 0.945 |
| EO | 0.10 (0.02, 0.18) | 0.09 (0.02, 0.12) | 0.553 |
| PT-S | 27.50 (21.90, 37.30) | 31.90 (28.40, 48.60) | 0.155 |
| PT% | 28.53 ± 13.38 | 24.68 ± 10.30 | 0.282 |
| INR | 2.29 (1.91, 3.07) | 2.84 (2.19, 4.40) | 0.179 |
| APTT | 50.70 ± 17.17 | 43.51 ± 10.10 | 0.080 |
| TT | 21.27 ± 5.29 | 19.75 ± 4.42 | 0.300 |
| AT-III | 27.50 (17.00, 58.20) | 31.00 (19.00, 67.00) | 0.732 |
| FDP | 14.28 (3.91, 32.39) | 7.20 (1.07, 26.06) | 0.227 |
| D-D | 67.87 (2.12, 1918.00) | 444.00 (157.00, 929.00) | 0.111 |
| FIB-C | 1.33 (1.02, 1.82) | 1.27 (0.82, 1.96) | 0.413 |
| ALT | 1088.00 (526.00, 2426.00) | 1192.00 (673.70, 2199.00) | 0.964 |
| AST | 1337.00 (354.00, 2261.95) | 695.00 (399.00, 1408.00) | 0.453 |
| TP | 56.66 ± 10.47 | 62.98 ± 6.63 | **0.016** |
| ALB | 32.62 ± 5.85 | 35.74 ± 5.48 | 0.078 |
| GLB | 24.20 (21.50, 27.20) | 28.80 (25.20, 30.00) | **0.021** |
| TBIL | 193.10 (120.80, 383.40) | 303.20 (140.48, 568.50) | 0.295 |
| DBIL | 130.30 (69.60, 232.00) | 131.40 (47.10, 363.30) | 0.759 |
| IBIL | 89.40 (35.00, 139.10) | 110.30 (42.15, 206.90) | 0.097 |
| CHE | 4007.00 (3210.00, 5430.00) | 3780.00 (3050.00, 6880.00) | 0.973 |
| ALP | 148.00 (122.00, 204.00) | 151.00 (111.00, 172.00) | 0.270 |
| GGT | 125.00 (50.00, 158.00) | 114.00 (59.00, 168.00) | 0.955 |
| TBA | 198.20 (110.34, 289.00) | 255.70 (129.09, 289.00) | 0.552 |
| GLU | 5.92 (3.90, 9.06) | 5.92 (4.20, 6.77) | 0.699 |
| TG | 3.04 ± 1.58 | 3.02 ± 1.14 | 0.961 |
| TC | 1.36 (0.62, 2.32) | 1.26 (0.92, 2.23) | 0.776 |
| HDL | 0.63 (0.31, 0.78) | 0.61 (0.50, 1.25) | 0.284 |
| LDL | 1.73 (1.07, 2.43) | 1.46 (1.12, 2.43) | 0.501 |
| K | 4.06 ± 0.73 | 3.72 ± 0.59 | 0.096 |
| Na | 137.24 ± 5.80 | 138.53 ± 2.87 | 0.321 |
| Cl | 101.50 (94.70, 105.50) | 102.80 (100.30, 105.30) | 0.601 |
| Urea | 4.25 (2.79, 9.22) | 3.62 (2.13, 5.76) | 0.195 |
| CRE | 79.00 (49.00, 113.00) | 53.80 (47.00, 70.00) | 0.088 |
| PCT | 1.19 (0.51, 3.31) | 0.93 (0.58, 1.57) | 0.785 |
| NLR | 5.93 (2.33, 12.40) | 4.68 (3.50, 10.31) | 0.733 |
| MLR | 0.50 (0.32, 1.09) | 0.75 (0.46, 0.95) | 0.219 |
| PLR | 108.12 (75.60, 198.75) | 133.33 (101.48, 244.30) | 0.246 |
| SII | 734.77 (381.12, 1144.00) | 858.27 (418.67, 1887.41) | 0.387 |
| SIRI | 3.57 (1.04, 7.56) | 5.02 (2.31, 8.32) | 0.453 |
