# Supplementary material for: Early peripheral monocyte dynamics are associated with 28-day mortality in acute liver failure: an exploratory longitudinal cohort study
Source: Front Med (Lausanne). 2026 Sep 10;13:1945730. doi: 10.3389/fmed.2026.1945730 (PMC13600835; doi:10.3389/fmed.2026.1945730)
Supplement: Supplementary file 9 [file Table4.docx]

**Table S4 Clinical characteristics and 28-day outcomes according to slope MONO tertiles**

| **Variable** | **T1 (lowest)** | **T2 (middle)** | **T3 (highest)** | **P value** |
| --- | --- | --- | --- | --- |
| 28-day mortality, n (%) | 5 (22.7%) | 7 (63.6%) | 11 (84.6%) | **0.001** |
| Gender | 10 (45.5%) | 1 (9.1%) | 9 (69.2%) | **0.012** |
| Age | 43.91 ± 17.12 | 47.64 ± 18.92 | 56.69 ± 17.22 | 0.126 |
| Ascites | 10 (45.5%) | 5 (45.5%) | 10 (76.9%) | 0.156 |
| Pleural effusion | 3 (13.6%) | 4 (36.4%) | 4 (30.8%) | 0.279 |
| Sepsis | 2 (9.1%) | 4 (36.4%) | 6 (46.2%) | **0.037** |
| Electrolyte disturbance | 16 (72.7%) | 7 (63.6%) | 10 (76.9%) | 0.764 |
| Gastrointestinal bleeding | 3 (13.6%) | 2 (18.2%) | 4 (30.8%) | 0.4626 |
| Respiratory tract infection | 4 (18.2%) | 4 (36.4%) | 6 (46.2%) | 0.1959 |
| Abdominal infection | 2 (9.1%) | 2 (18.2%) | 3 (23.1%) | 0.5124 |
| Renal failure | 4 (18.2%) | 4 (36.4%) | 9 (69.2%) | **0.0103** |
| Respiratory failure | 4 (18.2%) | 5 (45.5%) | 9 (69.2%) | **0.0101** |
| ICU | 1 (4.5%) | 5 (45.5%) | 5 (38.5%) | **0.0120** |
| Mechanical ventilation | 2 (9.1%) | 4 (36.4%) | 7 (53.8%) | **0.0140** |
| Vasopressor use | 2 (9.1%) | 5 (45.5%) | 5 (38.5%) | **0.0394** |
| CRRT | 1 (4.5%) | 2 (18.2%) | 6 (46.2%) | **0.0111** |
| Artificial liver support therapy | 15 (68.2%) | 6 (54.5%) | 7 (53.8%) | 0.6226 |
| WBC | 6.50 (5.28, 8.32) | 8.30 (6.05, 10.65) | 13.10 (8.20, 15.50) | **0.0293** |
| RBC | 4.42 (4.12, 5.23) | 3.82 (3.73, 4.12) | 4.28 (3.85, 4.96) | 0.1103 |
| HGB | 137.93 ± 20.15 | 123.73 ± 17.76 | 133.00 ± 26.65 | 0.2189 |
| HCT | 39.44 ± 7.14 | 37.05 ± 5.34 | 39.66 ± 8.19 | 0.6008 |
| MCV | 87.59 ± 8.55 | 90.53 ± 8.32 | 92.97 ± 7.73 | 0.1804 |
| MCH | 30.46 ± 3.51 | 30.23 ± 2.96 | 30.33 ± 3.62 | 0.9815 |
| MCHC | 345.00 ± 15.61 | 334.09 ± 10.39 | 335.62 ± 10.12 | **0.0418** |
| RDW | 15.00 (13.68, 16.80) | 15.20 (13.50, 17.70) | 17.70 (14.30, 18.10) | 0.1917 |
| SD | 45.01 ± 12.94 | 49.42 ± 13.92 | 48.96 ± 21.95 | 0.6848 |
| PLT | 135.50 (108.00, 201.25) | 158.00 (101.50, 193.00) | 99.00 (62.00, 150.00) | 0.2895 |
| PDW | 15.12 ± 2.43 | 16.08 ± 2.88 | 14.08 ± 2.86 | 0.1946 |
| PLT-PCT | 0.17 (0.12, 0.21) | 0.18 (0.12, 0.19) | 0.11 (0.07, 0.14) | 0.1219 |
| MPV | 11.46 ± 1.18 | 11.30 ± 1.35 | 10.35 ± 1.05 | **0.0299** |
| LYMPH | 1.15 (0.80, 1.45) | 1.54 (0.83, 1.66) | 0.94 (0.55, 1.97) | 0.6188 |
| MONO | 0.72 (0.64, 0.87) | 0.70 (0.54, 0.73) | 0.65 (0.40, 1.26) | 0.8791 |
| NEUT | 4.49 (2.90, 6.63) | 4.73 (3.55, 8.63) | 7.98 (5.57, 13.07) | **0.0277** |
| EO | 0.10 (0.02, 0.15) | 0.05 (0.01, 0.10) | 0.10 (0.02, 0.20) | 0.6629 |
| PT-S | 29.45 (23.20, 46.38) | 25.90 (21.80, 31.45) | 36.10 (27.50, 62.30) | 0.3467 |
| PT% | 30.50 (18.50, 36.95) | 32.80 (22.30, 39.00) | 21.70 (12.00, 31.00) | 0.2398 |
| INR | 2.49 (2.00, 4.15) | 2.00 (1.81, 2.92) | 3.05 (2.29, 3.32) | 0.3033 |
| APTT | 42.72 ± 9.17 | 49.71 ± 19.12 | 55.63 ± 17.36 | **0.0450** |
| TT | 20.06 ± 4.22 | 23.66 ± 5.85 | 19.31 ± 4.79 | 0.0695 |
| AT-III | 27.50 (18.17, 47.12) | 38.00 (20.70, 61.00) | 27.00 (13.20, 62.00) | 0.7392 |
| FDP | 6.83 (1.34, 26.06) | 17.72 (7.74, 39.80) | 14.09 (6.20, 32.39) | 0.3661 |
| D-D | 317.00 (1.90, 840.25) | 170.00 (3.98, 1085.00) | 506.00 (11.11, 1964.00) | 0.6194 |
| FIB-C | 1.44 (1.05, 1.98) | 1.16 (0.97, 1.32) | 1.64 (0.91, 2.30) | 0.3202 |
| ALT | 1245.17 (753.75, 2151.50) | 1080.90 (449.50, 2058.50) | 1042.00 (330.00, 3592.00) | 0.8475 |
| AST | 1002.23 (426.00, 2103.75) | 1343.60 (511.00, 1699.00) | 1216.00 (306.00, 2304.00) | 0.9814 |
| TP | 60.85 (58.35, 65.58) | 56.60 (51.85, 58.30) | 60.40 (45.90, 65.70) | 0.1260 |
| ALB | 35.34 ± 4.94 | 32.33 ± 5.03 | 32.35 ± 7.49 | 0.2252 |
| GLB | 26.86 ± 4.99 | 24.87 ± 4.34 | 23.48 ± 9.54 | 0.3219 |
| TBIL | 304.25 (153.08, 528.05) | 176.60 (115.95, 384.10) | 158.70 (101.80, 288.10) | 0.4310 |
| DBIL | 166.95 (86.83, 303.05) | 219.10 (81.10, 260.10) | 90.90 (54.40, 150.50) | 0.5650 |
| IBIL | 109.75 (49.38, 197.30) | 84.00 (29.55, 129.65) | 89.40 (35.00, 181.80) | 0.2076 |
| CHE | 4782.50 ± 2146.28 | 3917.09 ± 1345.26 | 4870.08 ± 1986.13 | 0.4129 |
| ALP | 149.50 (114.75, 173.05) | 160.00 (131.50, 223.50) | 143.00 (112.00, 204.00) | 0.5391 |
| GGT | 124.48 ± 75.95 | 96.36 ± 67.29 | 154.23 ± 110.88 | 0.2638 |
| TBA | 272.35 (129.09, 313.75) | 198.20 (76.70, 255.70) | 144.90 (110.34, 289.00) | 0.4846 |
| GLU | 5.03 (3.74, 6.33) | 6.86 (5.78, 8.70) | 7.56 (2.88, 13.82) | 0.1481 |
| TG | 2.96 ± 1.09 | 3.23 ± 1.78 | 2.98 ± 1.68 | 0.8681 |
| TC | 1.31 (0.96, 2.23) | 0.94 (0.54, 1.46) | 2.23 (1.23, 2.81) | 0.0615 |
| HDL | 0.61 (0.48, 0.87) | 0.40 (0.29, 0.72) | 0.74 (0.40, 0.90) | 0.3207 |
| LDL | 1.62 ± 0.73 | 2.00 ± 0.77 | 1.75 ± 0.90 | 0.4375 |
| K | 3.74 ± 0.57 | 4.19 ± 0.81 | 4.05 ± 0.73 | 0.1626 |
| Na | 138.77 ± 2.98 | 138.36 ± 7.03 | 135.38 ± 5.09 | 0.1271 |
| Cl | 102.93 ± 4.55 | 102.25 ± 7.09 | 97.41 ± 9.13 | 0.0636 |
| Urea | 3.72 (2.37, 4.78) | 4.44 (2.54, 11.10) | 8.25 (3.83, 12.59) | 0.1956 |
| CRE | 54.75 (47.00, 69.25) | 85.00 (47.50, 97.20) | 95.00 (65.00, 141.00) | 0.0516 |
| PCT | 0.94 (0.53, 2.98) | 0.66 (0.41, 0.91) | 1.99 (1.19, 3.96) | **0.0463** |
| NLR | 4.18 (2.24, 8.99) | 5.02 (2.34, 8.84) | 7.41 (5.93, 16.00) | 0.1912 |
| MLR | 0.64 (0.44, 0.93) | 0.56 (0.31, 0.89) | 0.58 (0.40, 1.75) | 0.7763 |
| PLR | 130.39 (99.64, 226.16) | 102.33 (69.77, 200.91) | 108.12 (58.93, 181.00) | 0.5862 |
| SII | 541.96 (304.50, 1515.29) | 577.56 (419.65, 1138.18) | 862.81 (734.77, 1264.00) | 0.6273 |
| SIRI | 3.09 (1.24, 6.88) | 3.57 (1.21, 6.33) | 6.64 (2.96, 10.09) | 0.4539 |
