# Supplementary material for: Early peripheral monocyte dynamics are associated with 28-day mortality in acute liver failure: an exploratory longitudinal cohort study
Source: Front Med (Lausanne). 2026 Sep 10;13:1945730. doi: 10.3389/fmed.2026.1945730 (PMC13600835; doi:10.3389/fmed.2026.1945730)
Supplement: Supplementary file 10 [file Table5.docx]

**Table S5 Pairwise DeLong comparisons of AUCs among prognostic models for 28-day mortality in acute liver failure**

| **Model 1** | **Model 2** | **AUC 1** | **AUC 2** | **Z** | **Unadjusted P value** | **Holm-adjusted P value** |
| --- | --- | --- | --- | --- | --- | --- |
| Baseline model | PTA model | 0.784 | 0.762 | 0.616 | 0.538 | 0.578 |
| Baseline model | MONO model | 0.784 | 0.858 | 1.393 | 0.164 | 0.491 |
| Baseline model | Combined model | 0.784 | 0.875 | 1.731 | 0.083 | 0.334 |
| PTA model | MONO model | 0.762 | 0.858 | 1.844 | 0.065 | 0.326 |
| PTA model | Combined model | 0.762 | 0.875 | 2.265 | **0.023** | 0.141 |
| MONO model | Combined model | 0.858 | 0.875 | 1.060 | 0.289 | 0.578 |
