# Supplementary material for: Early peripheral monocyte dynamics are associated with 28-day mortality in acute liver failure: an exploratory longitudinal cohort study
Source: Front Med (Lausanne). 2026 Sep 10;13:1945730. doi: 10.3389/fmed.2026.1945730 (PMC13600835; doi:10.3389/fmed.2026.1945730)
Supplement: Supplementary file 11 [file Table6.docx]

**Table S6 Linear mixed-effects models for early MONO trajectories during the first 3 days after admission**

|  | **Model 1 (95% CI)** | **P value** | **Model 2 (95% CI)** | **P value** | **Model 3 (95% CI)** | **P value** |
| --- | --- | --- | --- | --- | --- | --- |
| Intercept | 0.722 (0.491, 0.954) | **<0.001** | 0.817 (0.383, 1.251) | **<0.001** | 0.638 (0.238, 1.038) | **0.002** |
| Day | -0.002 (-0.006, 0.003) | 0.403 | -0.002 (-0.006, 0.003) | 0.403 | -0.002 (-0.005, 0.002) | 0.417 |
| Outcome | 0.084 (-0.233, 0.402) | 0.598 | 0.192 (-0.176, 0.560) | 0.302 | 0.032 (-0.306, 0.369) | 0.851 |
| Day × Outcome | 0.009 (0.003, 0.016) | **0.005** | 0.009 (0.003, 0.016) | **0.006** | 0.008 (0.003, 0.014) | **0.004** |

Model 1: Age

Model 2: Age + Gender

Model 3: Age + Gender + WBC
