# Supplementary material for: Early peripheral monocyte dynamics are associated with 28-day mortality in acute liver failure: an exploratory longitudinal cohort study
Source: Front Med (Lausanne). 2026 Sep 10;13:1945730. doi: 10.3389/fmed.2026.1945730 (PMC13600835; doi:10.3389/fmed.2026.1945730)
Supplement: Supplementary file 12 [file Table7.docx]

**Table S7 Multivariable logistic regression analyses of 72h-MONO-derived indicators and exploratory trajectory-informed clusters for 28-day mortality**

| **Exposure** | **Model 1** | **Model 2** | **Model 3A** | **Model 3B** | **Model 3C** | **Model 3D** | **Model 3E** | **Model 3F** |
| --- | --- | --- | --- | --- | --- | --- | --- | --- |
| MONO | 1.106  (0.960, 1.315), P = 0.197 | 1.272  (1.021, 1.751), P = 0.077 | 1.215  (0.961, 1.727), P = 0.186 | 1.393  (1.043, 2.098), P = 0.063 | 1.264  (0.924, 1.899), P = 0.197 | 1.345  (1.011, 1.996), P = 0.094 | 1.728  (1.193, 2.949), **P = 0.014** | 1.307  (0.969, 1.943), P = 0.121 |
| Delta MONO | 1.151  (1.018, 1.351), **P = 0.048** | 1.245  (1.046, 1.600), **P = 0.034** | 1.267  (1.043, 1.716), **P = 0.047** | 1.228  (1.019, 1.630), P = 0.069 | 1.227  (1.014, 1.655), P = 0.078 | 1.267  (1.045, 1.695), **P = 0.040** | 1.245  (1.036, 1.648), **P = 0.047** | 1.249  (1.023, 1.656), **P = 0.050** |
| Slope MONO | 1.027  (0.988, 1.077), P = 0.217 | 1.041  (0.993, 1.102), P = 0.109 | 1.038  (0.985, 1.111), P = 0.192 | 1.028  (0.976, 1.096), P = 0.328 | 1.030  (0.979, 1.097), P = 0.253 | 1.040  (0.988, 1.108), P = 0.148 | 1.034  (0.984, 1.097), P = 0.197 | 1.036  (0.980, 1.100), P = 0.186 |
| Cluster 2  vs  Cluster 1 | 0.255  (0.049, 1.049), P = 0.073 | 0.233  (0.030, 1.356), P = 0.124 | 0.227  (0.027, 1.456), P = 0.134 | 0.289  (0.030, 2.076), P = 0.235 | 0.246  (0.027, 1.743), P = 0.174 | 0.251  (0.033, 1.530), P = 0.146 | 0.293  (0.033, 2.024), P = 0.229 | 0.240  (0.020, 2.025), P = 0.206 |

Model 1: Raw model

Model 2: Age + Gender

Model 3A: Age + Gender + WBC + MELD

Model 3B: Age + Gender + NLR + MELD

Model 3C: Age + Gender + Sepsis + MELD

Model 3D: Age + Gender + WBC + PTA

Model 3E: Age + Gender + NLR + PTA

Model 3F: Age + Gender + Sepsis + PTA
