# Supplementary material for: Early peripheral monocyte dynamics are associated with 28-day mortality in acute liver failure: an exploratory longitudinal cohort study
Source: Front Med (Lausanne). 2026 Sep 10;13:1945730. doi: 10.3389/fmed.2026.1945730 (PMC13600835; doi:10.3389/fmed.2026.1945730)
Supplement: Supplementary file 13 [file Table8.docx]

**Table S8 Clinical and laboratory characteristics according to 72-h delta MONO status**

| **Variable** | **Delta MONO > 0** | **Delta MONO ≤ 0** | **P value** |
| --- | --- | --- | --- |
| 28-day mortality, n (%) | 17 (73.9%) | 5 (26.3%) | **0.002** |
| Gender | 11 (47.8%) | 9 (47.4%) | 0.976 |
| Age | 53.13 ± 16.57 | 42.89 ± 17.44 | 0.060 |
| Ascites | 15 (65.2%) | 8 (42.1%) | 0.134 |
| Pleural effusion | 6 (26.1%) | 4 (21.1%) | 1.000 |
| Sepsis | 8 (34.8%) | 3 (15.8%) | 0.291 |
| Electrolyte disturbance | 18 (78.3%) | 14 (73.7%) | 1.000 |
| Gastrointestinal bleeding | 5 (21.7%) | 4 (21.1%) | 1.000 |
| Respiratory tract infection | 9 (39.1%) | 5 (26.3%) | 0.381 |
| Abdominal infection | 4 (17.4%) | 3 (15.8%) | 1.000 |
| Renal failure | 12 (52.2%) | 5 (26.3%) | 0.089 |
| Respiratory failure | 13 (56.5%) | 5 (26.3%) | 0.049 |
| ICU admission | 8 (34.8%) | 2 (10.5%) | 0.083 |
| Mechanical ventilation | 10 (43.5%) | 3 (15.8%) | 0.053 |
| Vasopressor use | 8 (34.8%) | 3 (15.8%) | 0.291 |
| CRRT | 7 (30.4%) | 2 (10.5%) | 0.149 |
| Artificial liver support therapy | 12 (52.2%) | 15 (78.9%) | 0.071 |
| WBC | 10.20 (7.20, 14.70) | 6.50 (5.55, 9.00) | **0.043** |
| RBC | 4.29 ± 0.83 | 4.61 ± 0.87 | 0.235 |
| HGB | 130.91 ± 23.71 | 137.49 ± 21.34 | 0.350 |
| HCT | 39.06 ± 7.24 | 39.06 ± 7.56 | 0.999 |
| MCV | 91.58 ± 8.84 | 87.84 ± 8.31 | 0.167 |
| MCH | 30.22 ± 3.49 | 30.17 ± 3.01 | 0.962 |
| MCHC | 335.35 ± 9.10 | 346.42 ± 16.20 | **0.013** |
| RDW | 15.20 (13.85, 17.70) | 15.70 (14.45, 17.45) | 0.879 |
| SD | 47.30 (42.55, 54.00) | 47.60 (42.50, 55.15) | 0.879 |
| PLT | 124.00 (82.50, 178.50) | 130.00 (104.00, 194.50) | 0.733 |
| PDW | 14.90 ± 3.14 | 14.88 ± 2.33 | 0.989 |
| PLT-PCT | 0.14 (0.08, 0.18) | 0.17 (0.11, 0.20) | 0.495 |
| MPV | 10.97 ± 1.22 | 11.39 ± 1.35 | 0.299 |
| LYMPH | 0.99 (0.61, 1.84) | 1.30 (0.75, 1.46) | 0.960 |
| MONO | 0.71 (0.45, 1.14) | 0.70 (0.64, 0.84) | 0.850 |
| NEUT | 7.41 (4.96, 10.59) | 4.71 (3.45, 6.58) | 0.081 |
| EO | 0.05 (0.02, 0.11) | 0.10 (0.02, 0.14) | 0.657 |
| PT-S | 29.20 (22.55, 38.70) | 30.90 (27.15, 51.55) | 0.553 |
| PT% | 23.20 (18.15, 34.30) | 28.00 (16.00, 36.45) | 0.771 |
| INR | 2.76 (1.94, 3.17) | 2.72 (2.09, 4.71) | 0.587 |
| APTT | 49.90 (38.25, 58.90) | 41.50 (36.65, 50.50) | 0.172 |
| TT | 20.41 ± 4.37 | 20.91 ± 5.15 | 0.741 |
| AT-III | 27.00 (16.90, 60.10) | 31.00 (17.45, 46.25) | 0.990 |
| FDP | 14.09 (3.91, 32.39) | 7.20 (1.42, 29.23) | 0.425 |
| D-D | 170.00 (5.85, 1947.00) | 295.00 (4.69, 800.50) | 0.930 |
| FIB-C | 1.32 (1.04, 2.05) | 1.44 (0.92, 1.89) | 0.677 |
| ALT | 1394.00 (483.50, 2447.00) | 1088.00 (635.35, 2104.00) | 0.723 |
| AST | 1343.60 (410.00, 2282.97) | 987.45 (327.00, 1590.50) | 0.433 |
| TP | 57.15 ± 11.69 | 61.16 ± 7.50 | 0.187 |
| ALB | 32.53 ± 6.41 | 35.67 ± 5.17 | 0.087 |
| GLB | 24.90 (21.95, 29.65) | 27.50 (23.80, 29.50) | 0.426 |
| TBIL | 176.60 (115.95, 335.75) | 321.20 (161.30, 502.70) | 0.165 |
| DBIL | 118.30 (68.20, 255.15) | 196.10 (65.05, 291.90) | 0.570 |
| IBIL | 84.00 (31.05, 139.95) | 109.20 (66.15, 202.10) | 0.079 |
| CHE | 4491.00 (3198.00, 6480.00) | 3490.00 (3050.00, 5840.00) | 0.604 |
| ALP | 143.00 (125.50, 201.50) | 132.00 (104.50, 163.50) | 0.126 |
| GGT | 145.00 (55.00, 165.00) | 84.00 (53.00, 132.40) | 0.272 |
| TBA | 251.50 (71.62, 289.00) | 235.80 (119.72, 305.50) | 0.657 |
| GLU | 7.56 (3.40, 13.13) | 5.36 (4.08, 6.59) | 0.211 |
| TG | 2.94 ± 1.65 | 3.21 ± 1.22 | 0.543 |
| TC | 1.36 (0.78, 2.32) | 1.37 (0.76, 2.27) | 0.859 |
| HDL | 0.71 (0.35, 0.90) | 0.63 (0.52, 1.07) | 0.511 |
| LDL | 1.71 (1.26, 2.56) | 1.71 (1.10, 2.40) | 0.569 |
| K | 3.96 ± 0.75 | 3.89 ± 0.69 | 0.765 |
| Na | 136.78 ± 5.63 | 139.26 ± 4.07 | 0.106 |
| Cl | 101.50 (91.05, 105.40) | 102.20 (100.65, 105.90) | 0.306 |
| Urea | 4.23 (2.44, 10.52) | 4.20 (3.38, 5.92) | 0.960 |
| CRE | 84.00 (47.50, 118.50) | 62.00 (52.90, 81.35) | 0.511 |
| PCT | 1.32 (0.59, 3.31) | 0.87 (0.46, 1.41) | 0.418 |
| NLR | 6.25 (3.81, 15.55) | 4.48 (2.64, 8.00) | 0.157 |
| MLR | 0.58 (0.37, 1.51) | 0.69 (0.44, 0.95) | 0.960 |
| PLR | 108.12 (69.91, 232.15) | 122.22 (87.31, 157.49) | 0.820 |
| SII | 768.51 (544.89, 1302.61) | 500.58 (275.43, 1482.48) | 0.337 |
| SIRI | 6.50 (2.64, 10.26) | 3.26 (1.52, 5.64) | 0.235 |
