# Supplementary material for: Early peripheral monocyte dynamics are associated with 28-day mortality in acute liver failure: an exploratory longitudinal cohort study
Source: Front Med (Lausanne). 2026 Sep 10;13:1945730. doi: 10.3389/fmed.2026.1945730 (PMC13600835; doi:10.3389/fmed.2026.1945730)
Supplement: Supplementary file 14 [file Table9.docx]

**Table S9 Sensitivity analysis of linear mixed-effects models for MONO trajectories after re-including the excluded patient**

|  | Model 1 (95% CI) | P value | Model 2 (95% CI) | P value | Model 3 (95% CI) | P value |
| --- | --- | --- | --- | --- | --- | --- |
| Intercept | 0.686 (0.485, 0.887) | **<0.001** | 0.834 (0.451, 1.217) | <**0.001** | 0.655 (0.314, 0.995) | **<0.001** |
| Day | -0.001 (-0.003, 0.001) | 0.420 | -0.001 (-0.003, 0.001) | 0.419 | -0.002 (-0.003, -0.000) | **0.050** |
| Outcome | 0.130 (-0.152, 0.412) | 0.359 | 0.237 (-0.089, 0.564) | 0.151 | 0.075 (-0.215, 0.366) | 0.606 |
| Day × Outcome | 0.005 (0.002, 0.009) | **0.003** | 0.005 (0.002, 0.009) | **0.003** | 0.006 (0.003, 0.009) | **<0.001** |

Model 1: Age

Model 2: Age + Gender

Model 3: Age + Gender + WBC
